# Engaging members of a UK Somali community in identifying dementia research priorities: an example of adapting James Lind Alliance methods

**DOI:** 10.1186/s40900-026-01002-3

**Published:** 2026-11-09

**Authors:** Becky Field, Kate Gridley, Josephine Reynolds, Daniel Blackburn, Lise Sproson, Sam Creavin, Mark Clowes, Sahra Abdi, Muse Jama, Alicia O’Cathain

**Affiliations:** 1 Health and Care Research Unit, Sheffield School for Health and Related Research (SCHARR), Division of Population Health, University of Sheffield, Regent Court Sheffield, Sheffield, S1 4DA UK; 2 Older Adults’ Social Care Research Group (OSCaR), NIHR School for Social Care Research, University of York, York, YO10 5ZF UK; 3 Deep End Research Alliance, Division of Population Health, The University of Sheffield, Regent Court, Sheffield, S1 4DA UK; 4 Sheffield Institute for Translational Neuroscience (SITraN), University of Sheffield, Sheffield, S10 2HQ UK; 5 NIHR HealthTech Research Centre Long Term Conditions (Devices for Dignity), Royal Hallamshire Hospital, Sheffield, S10 2JF UK; 6 Centre for Academic Primary Care, Bristol Medical School, University of Bristol, University of Bristol, Bristol, BS8 2PS UK; 7 Technological University of the Shannon (Midlands), University Rd, Athlone, Co., Westmeath, N37 HD68 Ireland; 8 Israac Somali Community Association, Sheffield, S11 8FP UK

**Keywords:** Research priorities, Priority-setting, Dementia, Brain health, Somali, Public engagement, Public involvement, Underserved, Community engagement, Research inclusion

## Abstract

**Background:**

Dementia affects memory, problem-solving, language and behaviour. Engaging the public, including minority communities, in setting research priorities supports relevant, equitable research. Some ethnic minority communities face increased dementia risk, and barriers to diagnosis and culturally-appropriate care. A UK James Lind Alliance (JLA) dementia research priority setting exercise reported limited involvement of ethnic minority communities. Therefore, this study aimed to engage UK Somali community members to identify their brain health and dementia research priorities and compare these with the JLA top priorities.

**Methods:**

In 2025, we adapted the JLA approach using qualitative, participatory methods. Our four-phase approach included: (1) an initial workshop to engage community members, listen to lived experiences, provide information and identify uncertainties about dementia and brain health. (2) a rapid evidence search based on uncertainties identified (3) a second workshop for prioritising (4) a final workshop to confirm a ‘top 10’. Uncertainties from Workshop 1 were analysed thematically and iteratively by an expert steering group. Votes for top priorities were counted. A focused content analysis highlighted rationales for priorities.

**Results:**

Thirty-two people of Somali heritage attended Workshop 1, 15 of these were selected to attend Workshops 2 and 3. The top 10 priorities spanned key domains: awareness and risk reduction (three priorities), facilitating diagnosis (two priorities), improvingliving with dementia, coping, and caregiving (five priorities). Overlaps with the JLA top 10 were: improving diagnosis access, enabling living well, good care for people with dementia, carer support. Priorities about improving awareness, culturally-informed interventions to promote brain healthy diets, and the impact of faith on people affected by dementia, had not been included in the JLA top 10.

**Conclusions:**

Uniquely, this study developed dementia and brain health research priorities with UK Somali community members, demonstrating that involving this community in dementia research priority-setting is possible. The additional dementia research priorities should be considered alongside the JLA priorities to inform dementia research and service improvement agendas. Our four-phase approach can be incorporated into JLA priority setting partnerships to enhance inclusion of ethnic minority communities.

**Supplementary Information:**

The online version contains supplementary material available at https://doi.org/10.1186/s40900-026-01002-3.

## Abstract (significance)

The problem

What we did

What we found

Why this matters

Dementia is a condition that gets worse over time, affecting memory, problem-solving, language and behaviour. Research should help everyone, but ethnic minority communities are often left out, adding to unfair differences in health. This is important for dementia research because some communities have a higher risk of getting dementia, and face barriers to diagnosis and care. A national project identified priorities for dementia research but included few people from ethnic minority communities.

We worked with a UK Somali community in Sheffield to find out what their priorities for dementia research were. This involved:

1. **A first workshop (of 32 people)**: listening to people’s questions about dementia and brain health

2. **Checking**: seeing if research had already answered their questions

3. **A second workshop**: voting on which priorities mattered most

4. **A final workshop**: agreeing a ‘top 10’ list of priorities

We selected 15 of the 32 people from the first workshop to attend the second and final workshop.

Some of the ‘top 10’ were similar to the national list, such as the need for better diagnosis, help for family carers, and improving life for people with dementia. But some were different, such as: raising awareness, supporting a Somali ‘brain healthy’ diet, and the role of faith in supporting people affected by dementia.

This project shows working with an ethnic minority community to find out their priorities for dementia research is possible. Funders should look at this priority list to make sure future dementia research helps everyone. The way we ran this project can help other researchers include communities who are usually left out.

**Supplementary Information:**

The online version contains supplementary material available at https://doi.org/10.1186/s40900-026-01002-3.

## Introduction

Dementia is a progressive condition affecting cognition, behaviour, and daily independence [1]. Approximately 55 million people live with dementia worldwide, 982,000 in the UK [2]. Dementia has substantial impacts for individuals, families and society, costing the UK economy £42.5 billion annually [2], About 250,000 people with dementia in the UK are from ethnic minority backgrounds [2], but remain under-represented in standard priority-setting exercises [3]. This highlights an urgent need to adapt research engagement methods to capture their specific voices and priorities.

Patient and public involvement (PPI) in research can be defined as research being carried out ‘with’ or ‘by’ members of the public rather than ‘to’, ‘about’ or ‘for’ them. The term ‘public’, is often used to include patients and potential patients, carers and people who use health and social care services and people from organisations representing people who use services [4]. PPI can occur throughout the research process (e.g. to help set research priorities, or research questions, support study design choices, develop data collection tools, support analysis and dissemination) [4]. Whilst PPI is now usually embedded within UK research studies, it often fails to represent underserved populations [5]. Utilising PPI in identifying research priorities is particularly important because the public, including ethnic minority communities, have a right to shape research agendas. Their input is needed to ensure researchers ask relevant questions [6, 7]. Ethnic minority communities often experience worse health outcomes compared to the general population, so involving them in research priority setting has the potential to help reduce inequities in health [7]. In a systematic review about research priority setting for Black and minority health, Iqbal et al. [7] included 17 studies, covering a range of health issues. Iqbal et al. [7] highlighted the lack of involvement of Black and minority communities in establishing research priorities, and the limited involvement of these groups in any stage of the prioritisation process.

It is particularly vital to include ethnic minority communities within dementia research [3, 8, 9], and priority setting for dementia research. This is because increased dementia risk exists for some ethnic minority communities. For example, dementia rates are approximately 22% higher amongst black people in the United Kingdom (UK), compared to white people [10], although such broad ethnic categories may obscure heterogeneity of dementia risk across minority communities [10, 11]. Barriers exist to dementia diagnosis and services in the UK, and other countries, as services may not be culturally appropriate [12–16], influencing dementia service experiences. Somali communities are part of the Black British community. Predominantly Muslim, they make up 0.3% of the usual resident population of England and Wales [17]. Limited dementia-focused research exists about UK Somali communities, although some research reports their difficulties with accessing health services more generally [18–20]. This indicates the importance of seeking to involve and engage them with dementia research. Yet Somali voices have not been included in dementia research prioritisation. In 2015, Kelly et al. [21] published dementia research priorities, based on a James Lind Alliance Priority Setting Partnership (JLA PSP), involving the public, staff, service users and people affected by dementia [21]. The James Lind Alliance (JLA) brings patients, carers and clinicians together in research priority setting exercises to prioritise unanswered questions, to inform research and funding agendas [22]. Whilst the dementia research JLA PSP attempted to involve people from ethnic minority backgrounds, this was limited [21]. It cannot be assumed the JLA PSP dementia research priorities reflect those of specific ethnic minority communities. Coproducing research priorities with members of specific communities can contribute to relevant research being undertaken, promoting equity and service improvements [23].

Given existing inequities in dementia research, services and care, we wanted to engage members of a UK Somali community in a dementia research prioritisation exercise. Within this exercise, we included the term ‘brain health’ along with ‘dementia’, in contrast to the JLA PSP which used only ‘dementia’. We did this because since 2015, increasing evidence about dementia risk and risk-reduction [24] has been published, and ‘brain health’ is a more accessible term when discussing dementia ‘prevention’ or risk-reduction with the public [25].

This study aimed to:

i. Identify dementia and brain health research topics that are the highest priority for members of a UK Somali community, adapting the JLA approach to engage members of this community.
ii. Compare those priorities with the 2015 JLA PSP top 10 priorities.
iii. Identify implications for inclusive dementia research.

## Methods

### Design

We adapted the JLA approach [26] to engage a seldom-heard from UK Somali community, who are underserved by dementia services and dementia research. The JLA approach involves (i) asking patients, carers, and healthcare professionals to identify unanswered health questions through surveys (ii) verifying the questions against existing research evidence, and (iii) prioritising research questions via interest-holder voting and consensus workshops into “Top 10” lists to inform research, service development and funding agendas. We recognised that a tailored approach was needed, based on knowledge of specific barriers and communities [27]. Previous work with this Somali community found limited health literacy and dementia awareness, mistrust of services, and barriers to accessing the dementia care pathway [28, 29]. Thus, we decided a survey was unlikely to engage members of this community.

The Somali community we worked with lives in a Northern UK city and includes older generations (some do not read in Somali or English, speak or understand English fluently) and younger generations born in Somali/Somaliland or the UK, who communicate fluently in English. Hence, we needed a method that was accessible to community members and helped to build trust. The Deep End Research Alliance [30] shared materials from their research prioritisation work with another marginalised community, which aided conceptualisation of our approach [31].

We were also guided by a good-practice checklist for health research priority setting [32]. We chose a qualitative, participatory approach [31, 33–36] as this facilitated responsiveness to participants’ needs and experiences, recognising community partners as experts in their community [37]. In particular, we (i) replaced the JLA approach of an initial survey to identify questions generated by interest-holders with workshops [38] in a community setting; (ii) replaced the JLA detailed evidence review with a rapid evidence briefing [39], and (iii) replaced the full priority-setting process with a smaller-scale exercise which engaged community members. These decisions were guided by two Somali Community Research Link Workers (CRLWs) [37], who were trusted members of the Somali community and our research team. They led recruitment; provided cultural brokerage, advice, and facilitation; and offered interpretation and translation support. We chose not to involve NHS or care staff as research participants, as they had already been involved within the JLA PSP.

We undertook a four-phase approach consisting of: (1) an initial workshop to identify uncertainties; (2) rapid evidence search and briefing; (3) a second workshop to discuss remaining uncertainties and conduct a prioritisation exercise; (4) a third workshop to confirm the final ‘top 10’ priorities.

All workshops were held at a Somali community centre, had Somali interpreters, and a Somali lunch was provided.

### Participatory approach

A collaborative relationship between a Somali community organisation and university researchers was central to our approach. Previous projects had helped build trust [36, 40]. The CRLWs were a man and a woman, both Somali born, both Somali and English speakers. They were employed via the community organisation.

The CRLWs received a study induction and were supported to complete mandatory university information governance training. They then completed a bespoke training package in basic research skills provided by the DEEP END Research Alliance (https://sites.google.com/sheffield.ac.uk/dera/home) which covered: principles of research, confidentiality, informed consent, differences between PPI and research, qualitative and quantitative research, the need for inclusive research, the link worker role, recruitment skills, group facilitation skills, open and closed questioning styles. They also received dementia awareness training run by Innovations in Dementia Community Interest Company (http://www.innovationsindementia.org.uk/) which involved a person living with dementia as a co-trainer. This covered what dementia is, how it affects people and ways to support people affected. Later in the project they also completed ‘Dementia Stars’ training, which covered dementia causes, experiences, and ways to support people with dementia. This was provided by an organisation commissioned by the local authority to provide dementia awareness training to its staff and the wider community (https://www.enrichmentfortheelderly.org/training) Representation of both genders was important to enable recruitment of both men and women, and for participants to feel comfortable to express themselves in discussions.

The project ran from September 2024-December 2025, the priority-setting process between January-May 2025. We have structured our reporting according to the JLA process as far as possible [21, 26].

### Management and scope

An interdisciplinary project management group (PMG) (*n* = 12) included three NHS dementia clinicians (two GP clinical academics with special interests in dementia and health equity, one consultant neurologist and memory service lead), the two CRLWs, the Somali community organisation manager, a public involvement specialist, a public health researcher, a social care researcher, and two health services researchers (Appendix 1). The PMG oversaw the project, helping guide design and analysis. The role of the PMG is detailed later when each phase of the research is described. The scope of the priority-setting exercise was adapted from Kelly et al. [21]. The initial scope included prevention, treatment, diagnosis and care of people with dementia. We agreed to allow additional relevant topics to be included, if identified. The scope did not include causes of dementia.

### Ethical considerations

Ethical approval for the study was given by the University of Sheffield ethics committee (Division of Population Health). Workshop 1 was held as a public involvement and engagement event, so we did not seek signed consent for attending and participating. We obtained verbal consent to audio-record discussions, to support researcher note taking. Signed informed consent was received from participants before both Workshop 2 and Workshop 3 began. To facilitate receiving this consent from participants with limited English proficiency and/or those who preferred to communicate in Somali, or those who may have had literacy issues, the CRLWs had talked through and translated the participant information sheet verbally, during recruitment conversations prior to Workshops 2 and 3. Then, they recapped on the day of each workshop and translated the consent form verbally as needed.

### Recruitment and sampling

Recruitment relied on CRLWs’ existing community relationships and informal outreach in Somali-language spaces (e.g. community cafes, leisure centres, mosques). A recruitment flyer and easy-read style participant information sheets were coproduced with the CRLWs. CRLWs advised that translation of these printed materials was not required, as participants who could read would read English, and social media posts from the community centre were in English. For those for whom reading in either language was difficult, they interpreted participant documents verbally in Somali.

We aimed to recruit purposive samples: 30 people for Workshop 1, with around 14 of these people then attending Workshops 2 and 3. We set a pragmatic target of 30 participants for Workshop 1, following PMG discussion. We agreed a guide target of 30 for Workshop 1 as realistic, likely to result in a mix of lived experiences and a suitable number for the research team to facilitate. For Workshops 2 and 3, we aimed for less participants (approximately 14), who had attended Workshop 1, split into gender-specific groups of seven, in order to facilitate active, focus-group-style discussions.

For all workshops, we aimed to recruit adults (aged 18 years+) of Somali heritage or birth with a balanced mix of genders; for Workshop 1, people over and under 65, for Workshops 2 and 3, people over and under 50. We wanted to involve under and over 65s in Workshop 1 given the impact of dementia on both age groups, including early onset dementia. Also, the CRLWs felt older people, either living with or without dementia, or their carers may be difficult to recruit, due to underdiagnosis, stigma, and potential language or literacy issues within the older generation, so it was necessary to be flexible with exact sample size and age groups. The purposive sample we aimed for was a ‘guide’ rather than a fixed recruitment target, for both age groups.

Then, for all workshops we also aimed to recruit at least some people from each of the following categories: people living with dementia or people worried about their memory; family carers or relatives of people living with dementia or memory concerns; people interested in brain health and dementia.

We included these groups as all are interest-holders in dementia research. However, we knew from previous work with this community, and from the CRLWs, identifying and recruiting people self-identifying as living with dementia was unlikely given the underdiagnosis and stigma experienced. It was also unclear whether family carers would identify themselves as caring for a relative with dementia, for similar reasons. This was supported by the view of the PMG neurologist and coauthor (DB) indicating that people from the Somali community were rarely seen in local memory services. For Workshops 2 and 3 we invited 17 people who had attended Workshop 1 (to then recruit at least 14 participants), some of whom were family carers and/or met one of the other criteria. We adapted the age range for Workshops 2 and 3 (i.e., to include people over and under 50 + years) in response to Workshop 1 recruitment, which had found recruiting over 65s difficult. Reasons for declining were not collected.

We opted to use familiar, easily understood language i.e. ‘worried about your memory’ when recruiting, due to limited dementia awareness and stigma in this community. We were advised by the CRLWs that few people in the community would identify as living with dementia. This was supported by from the view of the PMG neurologist and coauthor (DB) indicating that people from the Somali community were rarely seen in local memory services.

We were concerned that workshops might discourage some people living with dementia or family carers from participating. So, we planned tailored, home-based interviews, as an alternative if suitable participants were identified.

All participants were offered shopping vouchers (£55) for their time and contribution.

### Phase 1 - identifying uncertainties (Workshop 1)

**Fig. 1:**
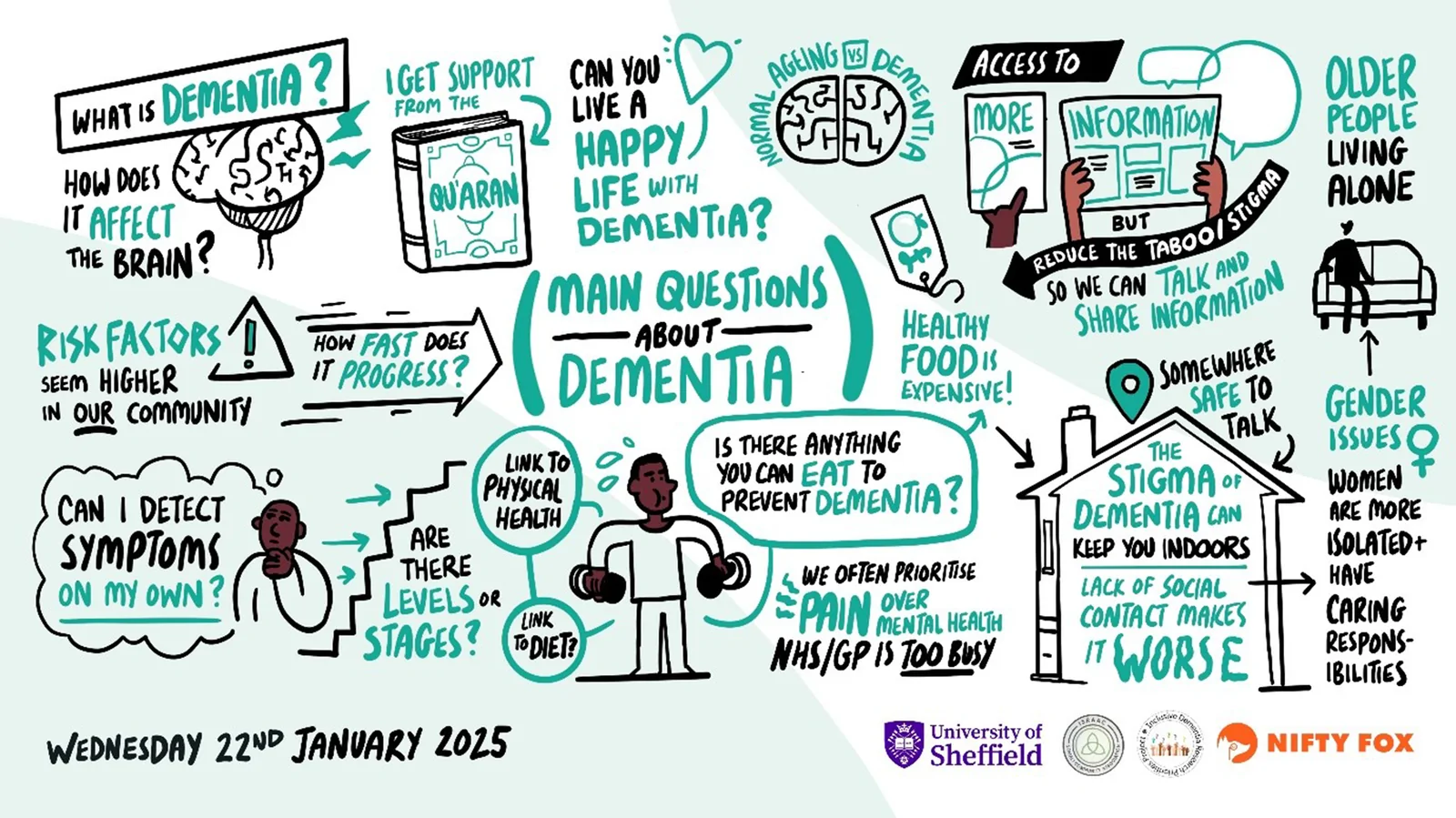
Visual summary of Workshop 1 (Phase 1) shared with participants

The JLA approach involves a survey to identify uncertainties; we adapted this to be a PPI workshop (i.e. Workshop 1). Workshop 1 lasted two hours. Three researchers gave an oral presentation with PowerPoint slides, presenting information about dementia, brain health, risk reduction, and where to get information and support, in English, with simultaneous Somali interpretation. Printed handouts with key service information published by the local authority were offered to attendees. Gender-specific seating was used, following CRLWs guidance. Four small discussion groups followed (two men’s groups, two women’s groups), facilitated by researchers. Participants were initially asked an open question: “What are your questions about dementia and brain health…?”. Then discussions were recorded via structured researcher note taking (using headings: ‘What’s important to us’; ‘Problems about dementia and brain health we are facing’; ‘What problems about dementia and brain health need solving’; ‘My community / my experience’) and summarised for participants via live visual scribing (Fig. 1).

Participants were encouraged to speak in Somali (with an interpreter in each discussion group) or English. The workshop was audio-recorded to aid typing of researcher notes post-workshop.

The first author (BF) collated all researcher notes from Workshop 1, reformatting them into drafts of researchable questions, and grouping them into themes. These were then shared with the PMG. The PMG used their expert knowledge to consider which questions were likely answerable through existing literature or guidelines. This approach was needed to help focus and limit the evidence search in Phase 2, and which questions to exclude because in their view, evidence already existed. For example, PMG members perceived that questions about risk factors and differences between dementia and normal aging were being widely studied. Non-researchable or out-of-scope questions were excluded (e.g. questions about to causes of dementia, or those where no underlying research question was indicated, such as comments about personal circumstances or requests for information, see Appendix 4 Table 8, for examples). Ambiguities were discussed and resolved. PMG agreed to include additional questions about limited dementia knowledge or awareness, stigma, isolation and access given that these were important topics for this community. We did not have predetermined criteria for making these decisions. It was unclear what research evidence existed on any topics within Somali communities specifically. Through an iterative process, the PMG drafted questions to share with the information specialist to guide the rapid evidence search in Phase 2.

### Phase 2: Verifying uncertainties (rapid evidence search and briefing)

The JLA [26] recommend summary questions agreed with a steering group be checked against existing evidence, ideally systematic review evidence, to ensure they are true uncertainties. They also acknowledge that alternative methods of evidence synthesis may be needed for some topics. We did not have the resources to systematically review evidence that might pertain to the reformatted questions identified from Phase 1. Therefore, for the purpose of evidence searching, the long list of questions resulting from Phase 1 was operationalised into a search strategy by an information specialist (MC) using the PCC (population, concept, context) (Appendix 2) framework recommended by the Joanna Briggs Institute [41] and converted into a set of prompts for a deep search Artificial Intelligence (AI) tool (Undermind.ai). Undermind offered the advantage of iterative searching that can prioritise a preferred term for one concept (e.g. Somali) before moving on to alternatives (e.g. African or Muslim) if the preferred term cannot be found, making it an efficient way to find the best available evidence for each question. Since Undermind launched in 2024, there have been a limited number of published evaluations [42, 43], published after we conducted our study (Feb-April 2025). Therefore, we also conducted a broad database search (Appendix 3) for all questions across MEDLINE, Embase and PsycINFO (via Ovid) to avoid missing relevant studies. The results retrieved by both sets of searches were screened for relevance by the information specialist (MC). Results were checked by the PMG to avoid omitting any known papers. Those closest to the eligibility criteria were selected for inclusion. No quality assessment was performed of included studies which is common for scoping reviews [44], and we had limited time and resources. While not a scoping review, our rapid evidence search had similar objectives, in that it aimed to map and summarize the evidence base for multiple questions identified from Workshop 1.

Review-level evidence was prioritised if available. A rapid evidence briefing was then compiled as a narrative summary describing the content of the included studies, then converted into a plain English summary using Google Gemini.

Prior to Workshop 2 in Phase 3, BF and AOC considered the rapid evidence briefing from Phase 2 and prepared a set of ‘remaining uncertainties’ as questions for Workshop 2 participants to prioritise.

### Phase 3: discussing remaining uncertainties and prioritising (Workshop 2)

Workshop 2 lasted two hours, facilitated by BF, KG and the CRLWs who interpreted as needed.

During Workshop 2 we presented evidence gaps, using accessible language and a visual summary. Participants then engaged in a structured prioritisation activity, in two groups (men, women), developed by the PMG and based on a Nominal Group Technique [26]. This involved each participant being given their own set of cards, with a question per card (based on the evidence gaps). Each set of question cards was the same for all participants. Contextual information was placed on the back of some cards to aid discussion and facilitation. Question cards were designed for accessibility with pictures and text in English and Somali. This aided the CLWs who were interpreting for some participants. Each participant was asked to select and speak uninterrupted about one to three of the questions they felt were most important. After everyone had spoken, and been listened to by the group, participants were asked to ‘vote’, individually, for the questions they felt were most important, by placing one coloured sticky dot on each of their own cards (green for ‘most important’ (green for ‘go’), orange for ‘unsure about these topics’, red for ‘least important (red for ‘stop’)’. Participants were also given a star sticker to mark a question card if they felt there was one particularly important question in the pack. Participants helped themselves to stickers placed on the tables. In the event, some participants did not make use of the red or orange stickers or place a dot on every card. Therefore, we counted up the questions that had the most green stickers and those marked with stars.

### Phase 4: confirming the final ‘top 10’ priorities (Workshop 3)

Two weeks later, Workshop 3 reconvened the same participants and lasted two hours, facilitated by BF, KG and the CRLWs who interpreted as needed. The purpose of Workshop 3 was to confirm or reject a ‘draft’ top 10 derived from the voting tallies from Workshop 2 and then refine wording and/or make adjustments based on consensus. In Workshop 3 we presented the ‘top 10’ priorities for research, derived from voting tallies from Workshop 2. In two groups (men, women) participants were asked to discuss whether they felt any key priorities were missing, and to discuss question wording. The top 10 priorities were displayed in large print on A4 cards, on a shared table or wall. We also asked participants to clarify the care settings that the care-related priorities referred to, and whether some priorities related to people with dementia and/or carers. After the gender specific discussions, we brought all participants back together to share what each group had discussed, and agreed the final wording and top 10 priorities via discussion. Finally, we asked for feedback on the whole prioritisation process. Again, the CRLWs interpreted as needed.

Workshops 2 and 3 were audio-recorded and transcribed.

### Analysis

A focused content analysis [45, 46] of Workshop 2 and 3 transcripts was completed by BF to enable the inclusion of some quotes in this paper. Analysis of Workshop 2 aimed to identify participant explanations of their reasons for priorities they selected to talk about as the most important. Analysis of Workshop 3 focused on participant views and responses to the top 10 questions presented.

## Findings

### Participants

Thirty-two people attended Workshop 1. Table 1 presents main characteristics and reason for attending. The age range was 28–70 years old. Most participants had been born in Somalia, and nine were carers of people with memory problems or dementia.

**Table 1:** Workshop 1 attendee characteristics and main reason for attending

	No. of attendees
**Gender**	
Men Women	16 16
**Occupation** ^1^	
Family Carer	11
Retired/pensioner	9
Paid employment	3
**Ethnicity** ^**2**^	
Black British	29
**Place of birth** ^**3**^	
Somalia/Somaliland	26
UK	2
**Main reason for attending** ^**4**^	
I care for someone with memory problems (or other ‘dementia like’ symptoms, e.g. changed thinking skills)	9
I care for someone with dementia	2
I am worried about my own memory or brain health	11
I am interested in brain health, memory and dementia	6
I care for someone with mild cognitive impairment	1
I am living with dementia	1

^1^missing data *n* = 7

^2^missing data *n* = 3

^3^missing data *n* = 3

^4^missing data *n* = 2

Fifteen of these 32 participants then attended both Workshops 2 and 3. Table 2 presents main characteristics and reason for participating. The age range was 30–69 years. Three participants were caring for someone with dementia or memory problems.

**Table 2:** Workshops 2 and 3 participant characteristics and main reason for participating

	Number of participants
**Gender​**	
Men Women	7 8
**Ethnicity** - Black British	15
**Country of birth**	
Somaliland or Somalia	14
UK	1
**Occupation** ^**1**^	
Family carer	5
Paid employment	6
**Main reason for participating**	
I care for someone with memory problems (or other ‘dementia like’ symptoms, e.g. changed thinking skills	2
I care for someone with dementia	1
I am worried about my own memory or brain health	3
I am interested in brain health, memory and dementia	6
I care for someone with mild cognitive impairment	1
Relative has dementia	1
Missing data	1

^1^ missing data *n* = 4

**Fig. 2:**
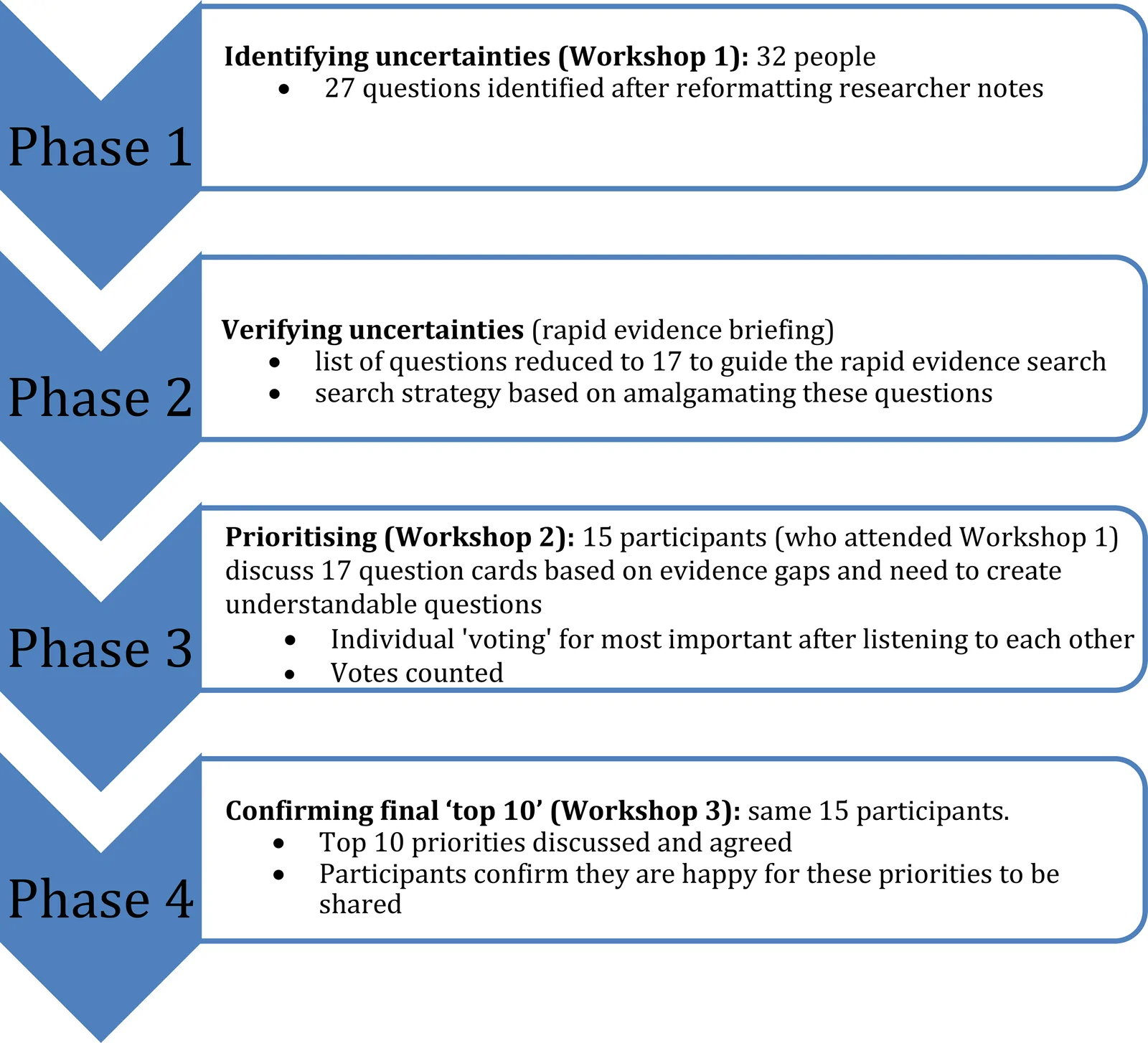
Summary of participant numbers and each study phase

Figure 2 summarises numbers of participants and each stage of the study.

### Phase 1: Identifying uncertainties (Workshop 1)

Researcher notes from Workshop 1 were combined and reformatted into 27 questions.

(Appendix 4). JLA PSP survey question themes of ‘Prevention’ ‘Diagnosis’ ‘Treatment’ and ‘Care’ [21] were applied where possible. We agreed to include additional initial themes: ‘Knowledge & awareness’; ‘Stigma & isolation’ and ‘Access’ given these were important topics for this community. Following PMG input, the draft questions were reworded and some combined to create 17 questions to guide the evidence search within Phase 2 (verifying uncertainties) (Appendix 5). Requests for information or comments about personal circumstances were removed, as per JLA guidance [17,p.47]. Some of these comments highlighted lack of knowledge about how to seek help for memory or dementia-related concerns, or feeling unable to, indicating support or information needs. We agreed to share this finding with local dementia service interest-holders (e.g. commissioners, service leads). Figure 1 presents the visual summary of Workshop 1 discussions.

### Phase 2: Verifying uncertainties (rapid evidence briefing)

The seventeen questions (Appendix 5) were shared with the information specialist, who used these to guide the rapid evidence search. Two were about prevention, four about access, one about diagnosis, one about treatment, two about knowledge and awareness, three about stigma and isolation, five about care. A rapid evidence briefing summarised and confirmed areas of remaining uncertainty (Appendix 6.1), and we produced a plain English version (Appendix 6.2) to aid development of a visual summary to use within for Workshop 2 to facilitate communication with participants about these areas of uncertainty (Appendix 6.3).

### Phase 3 discussing remaining uncertainties and prioritising (Workshop 2)

Seventeen questions were presented on cards for prioritising in Workshop 2 (Appendix 7). The process of identifying the questions for Phase 3 was driven by the need to communicate questions in a way participants could understand and to create questions for prioritising from the evidence briefing. Questions were about: awareness of dementia and risk (seven questions - four about dementia awareness, awareness of risks and risk reduction, three about diet and brain health/risk reduction); dementia risk assessment (three questions); getting help with memory problems or dementia diagnosis (two questions); living with dementia, caring and coping (five questions - two about living well with dementia and what good care could mean, one about support for family carers, one about the role of faith).

### Phase 4: confirming the final ‘top 10’ priorities (Workshop 3)

The top 10 priorities identified after counting the ‘votes’ from Workshop 2 were shared in Workshop 3 (Appendix 8). Some changes were suggested e.g. amalgamating questions about brain health and Somali food, and consensus obtained to do this. Facilitators had suggested that the question ‘What support do Somali family carers of people living with dementia need to prevent them feeling isolated?’ could be amended to include both family carers and people living with dementia, but some women participants felt it important to have separate questions for each, and when this was taken back to the whole group, all agreed. Participants agreed that ‘care’ (Question 7, Table 3) should be understood as care in hospital, at home, care packages, and in community settings. Once these changes were made, all participants were happy for the ‘top 10’ to be shared publicly. Table 3 presents the final top 10 priorities, after these changes were made.

**Table 3:** Final ‘top 10’ priorities (not in any order of priority)

1. How do people in the UK Somali community understand dementia and brain health?
2. How can people in the UK Somali community be supported to become more aware of what dementia is?
3. Can culturally-informed interventions be developed to promote a brain healthy diet with the Somali community?
4. How can services make it easier for people from the Somali (or other minority communities) to see a specialist to find out if they have dementia or not?
5. How can the UK Somali community (or other minority communities) be supported to see a specialist to find out if they have dementia or not?
6. What does living well (good quality of life) with dementia mean to people in the UK Somali community?
7. What would good care for people living with dementia in the UK Somali community look like?
8. What support do Somali family carers of people living with dementia need to prevent them feeling isolated?
9. What support do Somali people living with dementia need to prevent them feeling isolated?
10. Can faith-based activities, or being part of a faith-based community reduce the impact of dementia on people with dementia, and their family?

(See Appendix 9 for the Workshop 3 visual summary)

### Evaluation

During Workshops 2 and 3, participants were asked if any key points were missing when shown the visual summaries. During Workshop 3, we sought feedback on participant experience, interpretation/translation, workshop format, and gender-specific groups. Overall feedback was positive, with some participants noting the value of discussing dementia together. The men’s group debated the challenges of translating ‘dementia’ into Somali; we had learnt during Workshop 1 that the common term was stigmatising, but no clear alternative was agreed. One participant highlighted the need for translating research findings into practice, which he felt was not addressed by the final priorities.

### Comparing final priorities from the JLA PSP and the final priorities identified in this project

Table 4 presents the top 10 priorities identified by the JLA PSP [21] and priorities identified in this study. There were areas of overlap and differences. These are highlighted below, with quotes from Workshop 2 to illustrate areas of difference between the two lists.

**Table 4:** Comparison of JLA PSP top priorities and top priorities from this study

JLA PSP top 10 priorities (Kelly et al. 2015)	A UK Somali community
-	How do people in the UK Somali community understand dementia and brain health? How can people in the UK Somali community be supported to become more aware of what dementia is? Can culturally informed interventions be developed to promote a brain healthy diet with the Somali community?
What are the most effective components of care that keep a person with dementia as independent as they can be at all stages of the disease in all care settings?	What does living well (good quality of life) with dementia mean to people in the UK Somali community? What would good care for people living with dementia in the UK Somali community look like? What support do Somali people living with dementia need to prevent them feeling isolated?
How can the best ways to care for people with dementia, including results from research findings, be effectively disseminated and implemented into care practice?	-
What is the impact of an early diagnosis of dementia and how can primary care support a more effective route to diagnosis?	How can services make it easier for people from the Somali (or other minority communities) to see a specialist to find out if they have dementia or not? How can the UK Somali community (or other minority communities) be supported to see a specialist to find out if they have dementia or not?
What non-pharmacological and/or pharmacological (drug) interventions are most effective for managing challenging behaviour in people with dementia?	-
What is the best way to care for people with dementia in a hospital setting when they have acute healthcare needs?	-
What are the most effective ways to encourage people with dementia to eat, drink and maintain nutritional intake?	-
What are the most effective ways of supporting carers of people with dementia living at home?	What support do Somali family carers of people living with dementia need to prevent them feeling isolated?
What is the best way to care for people with advanced dementia (with or without other illnesses) at the end of life?	-
When is the optimal time to move a person with dementia into a care home setting and how can the standard of care be improved?	-
What are the most effective design features for producing dementia friendly environments at both the housing and neighbourhood levels?	-
-	Can faith-based activities, or being part of a faith-based community reduce the impact of dementia on people with dementia, and their family?

#### Common priorities

Both lists included questions about improving care and supporting people to live well with dementia. Both exercises identified supporting family carers as key. This suggests alignment in recognising the importance of supporting people with dementia and carers within the community to live as well as possible. Both lists had questions about increasing support for diagnosis.

#### Priorities this study identified, which the JLA PSP did not

Faith was not referenced within the JLA PSP top 10, nor within their long list of questions [12-Supplementary file A]. In this study, there was discussion in all workshops about the benefits participants gained from their Muslim faith and practices, and potential dementia-related research on this topic. For example, whether learning the Qu’ran or prayer routines could prevent cognitive decline, the impact of Mosque attendance and faith on people affected by dementia. The following quotes are from Workshop 2, when participants talked about why they selected the question *‘Can faith-based activities or being part of a faith-based community*,* reduce the impact of dementia on people with dementia*,* and their family carers?*’ as one of the most important. These quotes illustrate interest in faith as a topic of importance for dementia research, and how participants associated benefits with their faith practices:

In the faith, it’s not individual. Most of the time, it’s five times a day of congregation in the mosque…helps people to come together and socialise… gets them out of isolation…(male participant 1).

….This [question] is very important. Because Somali people, we are religious….reading the Qur’an…affect[s] our brain…we are learning a lot. We are reading. We are also understanding. So this can affect our brain, can function…so that is a good question (male participant 2).

In this study’s top 10, three questions focused on dementia awareness and brain health. The JLA PSP top 10 did not include questions about these issues and none of the prevention questions from their longer list made it into their top 10 [21]. The following quotes illustrate how some participants acknowledged limited risk reduction knowledge relating to diet, dementia awareness and stigma within their community, when discussing their top priority questions in Workshop 2:

I’ll go for the first question ‘How do people in the UK Somali communities understand dementia and brain health’ Actually, their understanding is very limited. They don’t have much information or awareness at all. But they have also bad names, or they have got taboo in their perception about dementia. (female participant 1).

I choose it [Question: ‘How could the UK Somali community be supported to eat a brain healthy diet? ] because…bad diet can increase your memory and dementia problem…we have a set… ingredient[s] we use in the Somali community…we don’t look for different, like, options…but our food most of times is the carbohydrate food. And we need that less… (female participant 5).

#### Priorities the JLA PSP identified, which this study did not

The JLA PSP top 10 included “challenging behaviours” while the top 10 from this study did not. This issue did not come up in our workshops and so did not enter our evidence search process and inform subsequent priorities. Discussing this topic in a workshop setting may have been too sensitive, but we do not know why it was not discussed. Similarly, the JLA PSP top 10 included a question about dementia-friendly environments, while this study did not. However, the importance of social environments and reducing isolation, via safe, accessible, welcoming community spaces for people with dementia and carers was discussed, although not framed in the language of “dementia-friendly environments”.

In contrast to the JLA PSP, questions about specific care settings were not included in this study’s top 10 e.g. hospital, residential or end of life care. However, the importance of improving care in different settings was discussed. For example, the needs of Somali people in hospitals were raised during Workshop 2, but questions relating to that topic had not been noted during Workshop (1) Some participants talked about different care settings, when explaining why they had selected the question *‘What would good care for people living with dementia in the UK Somali community look like?’* as a priority in Workshop (2) The following quotes highlight concerns about isolation of Somali people with dementia in hospital, of elders within community settings, and cultural expectations for home-based care:

…in hospital care…lack of understanding of the language, not having community support there, or someone [who] can interpret or make some activity groups...not understanding the doctors or the nurses…maybe providing a…dementia champion in every community would help… (female participant 6).

…how to create an area where the elderly people can come together…Because most of them are staying at home, isolated…. (male participant 1)

…His father’s been neglected…same with my uncle… where he’s deteriorated…it’s just towards the end, they wanted to do something, but it’s just too late…then they wanted to say…how do you want your father to be cared for? Whether to be in the hospitals or in a nursing care? And in our culture, we don’t like nursing care or hospitals. We like keeping it within our house and within our families…back home, we’ve got massive extended families… (male participant 7).

Workshop 2 and 3 discussions also suggested that religious and cultural beliefs made discussing death particularly sensitive. This may have led to topics relating to hospital or end-of-life care being underrepresented in Workshop 1 and therefore not included in the evidence briefing.

Finally, JLA PSP had a top 10 priority about disseminating and implementing research evidence into practice whereas this study did not. However, in later workshops some participants asked how the questions presented could lead to changes in practice, suggesting the importance of this topic to participants.

## Discussion

The top 10 priorities identified in this study supplement those identified by the UK JLA PSP. They covered key domains, including awareness, understanding, diet and risk reduction, diagnosis, living with dementia, coping, and caregiving. Topics of overlap with the JLA PSP were diagnosis, living well with dementia, care and carer support. This indicates alignment in recognising the importance of living well and supporting people with dementia and family carers within the community. However, some priorities differed for this UK Somali community. The role of faith in supporting people affected by dementia, improving dementia awareness and risk reduction through diet, were research priorities for this community, prompting much discussion within the workshops. The question about the role of faith was often selected as a question people chose to talk about as being one of the most important in Workshop 2, and participants appeared pleased it had made it into the Top 10 in Workshop 3. This seemed to be because participants felt their faith to be central to their lives, their community and to coping with dementia. This central role of faith reflects qualitative evidence highlighting faith as both a coping mechanism and a framework for understanding health amongst Muslim and refugee communities in the UK and elsewhere [47–50]. In the UK, Kevern et al.’s [48] qualitative study reported faith supported dementia family carer resilience amongst a British Pakistani Muslim community, and found that the wider faith community and its leaders did not typically offer support, suggesting this perhaps inadvertently contributed to delayed help-seeking or stigma, likely due to limited dementia awareness among religious leaders and the wider community. A scoping of review of faith-driven health interventions in several countries found faith-based approaches effective in improving health outcomes, promoting positive health behaviours, addressing communication and cultural barriers [49]. A review about measures of religion and spirituality in dementia also reports positive associations between religion and health [50]. However, faith-tailored interventions for people living with and dementia and their families from minority ethnic communities remain under-researched [49, 50].

Participants discussed how dementia is often not openly discussed or disclosed within their community. This finding, and the final priorities agreed within this study about improving brain health, dementia awareness and reducing stigma aligns with other evidence highlighting dementia stigma, the importance of improving dementia awareness and diagnosis access amongst other ethnic minority groups, in the UK and internationally [3, 12, 15, 51–56]. For example, focus group research with British Bangladeshi communities highlighted gaps in dementia knowledge [56]. A systematic review of interventions to promote dementia knowledge among ethnic minority groups, mostly in the USA, found most demonstrated acceptability and showed promise of positive outcomes, but also that evaluation of such interventions is still developing [54]. Patel et al. [55] reports that dementia research priorities identified by interest-holders in the USA, Canada and the UK all demonstrated demand for post-diagnostic community support and family education.

Methodologically, our use of Community Research Link Workers (CRLWs) and collaboration with a Somali community organisation operationalises recommendations for more inclusive dementia research [51].

### Reflections on adapting the JLA approach

It was necessary to adapt the JLA approach to facilitate inclusion of people from an underserved community. On reflection, we think our qualitative participatory approach led to successful engagement of community members and recommend this approach is adopted within the JLA, as part of their priority setting exercises to increase inclusion of ethnic minority communities. Beginning the process with a public engagement and involvement event (Workshop 1) was important to help create interest in the project, build trust and share dementia related information with community members and for people affected by dementia, given we knew from the CRLWs, the community organisation and previous projects that understanding about dementia was limited and it was stigmatised.

Our methods will have influenced what participants felt comfortable sharing, thus influencing what was included within the rapid evidence briefing and subsequent priorities. For example, the emphasis on faith and culturally appropriate care likely stemmed from the dialogue enabled by our approach but it may also have constrained discussion of topics like end-of-life care or ‘challenging behaviours’. Perhaps such topics were less likely to be raised in group settings, but this remains unclear.

Throughout we reflected on how we could stay close to JLA guidance or how much we needed to adapt it. For example, JLA suggest removing comments or questions about awareness of dementia, and in their dementia PSP passed such questions to the Alzheimer’s Society [21]. Yet we identified dementia awareness and stigma as important themes within this community so decided to keep these themes within scope. We also shared this finding with local dementia service commissioners. In addition, the JLA PSP used the term ‘prevention’ in their survey, whereas we used the terms ‘brain health’ and talked about reducing risks rather than prevention in our study. This highlights the importance of methodological flexibility when working with communities underserved by mainstream services and underrepresented in research [5, 35].

Our study highlights the need for researchers and services to use accessible language, and not assume shared understandings of dementia, risk reduction, what research is, or support services. For example, some terminology used within the JLA PSP top 10 dementia research priorities would have been unhelpful in this study (e.g. ‘challenging behaviours’; ‘primary care’; ‘effective components’ ‘disseminated’). Engaging in discussion about research priorities was unfamiliar for some participants, but with our adaptations most talked ably about their health and care priorities in ways relevant to research. We also felt we had a responsibility to link people to appropriate services, rather than ‘signposting’ alone, which we also did (e.g. advising participants contact their GP, providing information leaflets), providing tailored follow-up support, via the CRLWs when needed (e.g. to access memory assessment, and a care package review).

### Strengths and limitations

This study is the first to identify dementia and brain health research priorities in partnership with members of a UK Somali community, to the best of our knowledge. Their priorities are important for dementia researchers, research funders and service improvement. This study demonstrates the feasibility and value of engaging Somali communities in shaping dementia research agendas, whilst also highlighting service gaps.

There were several limitations. Firstly, meaningful inclusion of people living with dementia was limited. We had planned for tailored interviews with people living with dementia but struggled to identify suitable participants. This may partly reflect evidence that some people from ethnic minority communities present to services at later stages of dementia [11, 57] inherently limiting the ‘pool’ of people with ‘early’ dementia suitable for participating in studies such as this. Second, despite discussing translation and interpretation needs with the CRLWs, we were not initially aware that the commonly used Somali term for dementia was stigmatising. We learnt this at the end of Workshop 1, during discussion with participants, as when CRLWs were interpreting they tended to use the stigmatising Somali word, as there is not a direct translation for the word dementia in Somali. During subsequent workshop discussions, no clear Somali alternative was agreed, and we agreed to just use the English word ‘dementia’ going forward. Third, time and resource constraints meant our evidence briefing was not comprehensive or quality assessed. Also, the rapid review was informed by a search strategy developed following subjective judgements of PMG members about areas of existing research, and thus which questions should guide topics included in the rapid review, this may have introduced researcher bias. The rapid evidence search was less robust than a comprehensive scoping review process, but it enabled us to perform a similar function, with the prior knowledge of PMG members used to mitigate the risk of missing relevant studies. We think we identified the breadth of research in this area. Fourth, iterative development of questions in Phase 1, generated from Workshop 1 researcher notes, and selection of questions for Workshop 2 in Phase 3 was necessarily subjective, although the PMG helped ensure multiple viewpoints contributed. Fifth, during Workshop 2 some participants did not follow all instructions i.e. to place a sticker on each card. This may have been because the task was too complex for some and time limitations. We found interpretation into Somali of the voting instructions and content on each card took longer than envisaged, despite preparation. There were also interruptions at the community centre, reflecting how when working in community settings researchers cannot always control the environment. Sixth, adhering to Viergever et al.‘s [32] health research priority setting checklist was challenging for two items; for ‘Evaluation’ we sought in-person feedback as we were mindful of participant burden, but this may have led to overly positive responses; for ‘Planning for implementation’ we have shared findings with service commissioners but for priorities to be implemented, funding needs to be secured, which we have yet to achieve. As Staley et al. [58] suggest to follow through on a Top 10, additional work is needed to refine priority topics further into research questions to be matched with appropriate funding sources. Finally, the focused content analysis was single-coded by BF, another coder may have identified different relevant illustrative quotes.

### Implications

Our methods can be used more widely to enable members of other underserved communities to become involved in dementia research, and we believe, is likely applicable to other topics within health and care research priority setting. We adapted the JLA approach because they had already identified dementia research priorities but acknowledge that other participatory approaches [6, 59] could be helpful when aiming to include underserved communities. As Rowbotham et al. [60] note, there is no ‘one-size-fits-all’ for patient and public involvement methods. We found working with CRLWs and a community organisation was essential, and such an approach has also been successful in other projects [29, 61, 62]. We recommend this approach as CRLWs provide trust and cultural brokerage to enable participation and engagement in the research process. Developing the research capacity and dementia awareness of community members as CRLWs means they can support future inclusive dementia research. Tracking potential outcomes, such as increased dementia knowledge, for example through pre/post knowledge assessments [63], may help measure the broader impacts of dementia research priority setting exercises undertaken in communities where limited dementia knowledge and stigma exist.

The identified priorities reflect the concerns of this specific Somali community, may resonate with other Somali communities and warrant exploration in other ethnic minority communities, such as Black African communities or communities with a strong religious focus. Exploring if our study’s priorities, such as the role of faith, resonate with other minority ethnic communities, would be worthwhile. Given the central role of faith in helping some individuals cope with the impacts of dementia, research that co-produces and evaluates faith-based dementia interventions is needed. To address the identified priorities surrounding brain health and dementia awareness in communities with oral traditions, literacy barriers, or preferences for visual based information, future studies could evaluate accessible visual or digital formats, such as YouTube or video platforms [64], changes in help-seeking attitudes [65], or dementia knowledge amongst Somali, and other ethnic minority communities.

Our study reflects a key tension in dementia research: balancing methodological rigour with inclusivity [66]. For example, we aimed to recruit people who self-identified as living with dementia, or with ‘memory problems’ or their family carers, as have others [16]. This was an imperfect ‘proxy’ for cognitive impairment or dementia. This illustrates the difficulty of applying standard eligibility criteria in communities where dementia is underdiagnosed, not openly discussed, or not understood in biomedical terms. In England, the National Institute of Health and Care Research requires applicants to address inclusion, equality and diversity [67] but it remains unclear how this is, or can be, applied within dementia research if dementia diagnosis or self-identification of dementia is required. Such inclusion criteria risk further excluding underserved populations. Given current practice lacks culturally competent diagnostic pathways, dementia research studies likely need to offer participants from underserved communities advocacy and support, including translation/interpretation to obtain assessment, diagnosis, and appropriate care, if desired. The top priorities identified by this study highlight that addressing barriers to diagnosis and care remains critical for equitable dementia service improvement.

## Conclusions

This study shows how dementia and brain health research priorities can be developed with members of a Somali community. Our approach can be included within JLA priority setting exercises to enhance inclusion of ethnic minority communities. The questions prioritised are wide ranging, covering raising dementia awareness, diet and brain health, diagnosis and care in all settings. Addressing these will require collaborative and inclusive approaches. As existing evidence highlights, other minoritised communities are also underserved by dementia services, yet are at increased risk of developing dementia. It is critical for researchers and funders to work collaboratively with such communities and involve them in research priority setting if we are to achieve equity within dementia research and services. The top 10 priorities identified in this study supplement those identified by the JLA PSP and should be considered alongside existing dementia research priorities.

## Supplementary Information

Below is the link to the electronic supplementary material.

Supplementary Material 1

Supplementary Material 2

Supplementary Material 3

Supplementary Material 4

Supplementary Material 5

Supplementary Material 6

Supplementary Material 7

Supplementary Material 8

Supplementary Material 9

Supplementary Material 10

Supplementary Material 11

## Data Availability

The datasets used and/or analysed during the study cannot be made publicly available due to risk of identifying participants and as we did not seek participant consent for this. The authors welcome contact from those interested in this study and materials used can be made available upon reasonable request to the corresponding author.

## Abbreviations

AI: Artificial Intelligence
CRLWs: Community Research Link Worker
GPs: General Practitioner
JLA: James Lind Alliance
PMG: Project Management Group
PSP: Priority Setting Partnership
