# Supplementary material for: Engaging members of a UK Somali community in identifying dementia research priorities: an example of adapting James Lind Alliance methods
Source: Res Involv Engagem. 2026 Nov 9;12:183. doi: 10.1186/s40900-026-01002-3 (PMC13749654; doi:10.1186/s40900-026-01002-3)
Supplement: Supplementary file 1 [file 40900_2026_1002_MOESM11_ESM.pdf]

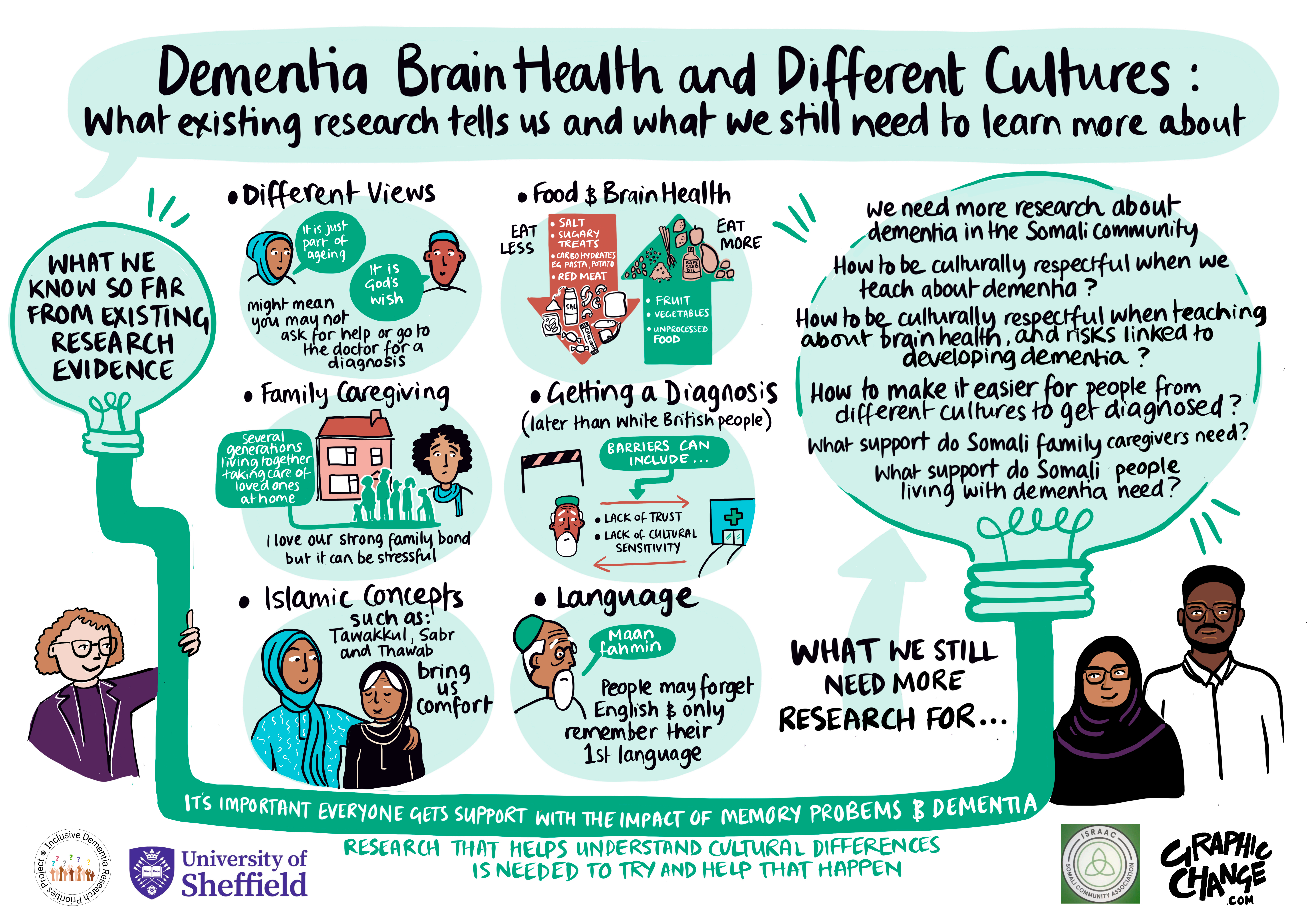
Dementia Brain Health and Different Cultures :
What existing research tells us and what we still need to learn more about
WHAT WE KNOW SO FAR FROM EXISTING RESEARCH EVIDENCE
• Different Views
It is just part of ageing
It is God's wish
might mean you may not ask for help or go to the doctor for a diagnosis
• Food & Brain Health
EAT LESS
• SALT
• SUGARY TREATS
• CARBOHYDRATES EG PASTA, POTATO
• RED MEAT
EAT MORE
• FRUIT
• VEGETABLES
• UNPROCESSED FOOD
• Family Caregiving
several generations living together taking care of loved ones at home
I love our strong family bond but it can be stressful
• Getting a Diagnosis
(later than white British people)
BARRIERS CAN INCLUDE...
• LACK of TRUST
• LACK of CULTURAL SENSITIVITY
• Islamic Concepts
such as: Tawakkul, Sabr and Thawab
bring us comfort
• Language
Maan fahmin
People may forget English & only remember their 1st language
We need more research about dementia in the Somali community
How to be culturally respectful when we teach about dementia?
How to be culturally respectful when teaching about brain health, and risks linked to developing dementia?
How to make it easier for people from different cultures to get diagnosed?
What support do Somali family caregivers need?
What support do Somali people living with dementia need?
WHAT WE STILL NEED MORE RESEARCH FOR...
IT'S IMPORTANT EVERYONE GETS SUPPORT WITH THE IMPACT OF MEMORY PROBEMS & DEMENTIA
RESEARCH THAT HELPS UNDERSTAND CULTURAL DIFFERENCES IS NEEDED TO TRY AND HELP THAT HAPPEN
Inclusive Dementia Research Priorities Project
University of Sheffield
ISRAAC SOMALI COMMUNITY ASSOCIATION
GRAPHIC CHANGE .COM
