# Supplementary material for: Engaging members of a UK Somali community in identifying dementia research priorities: an example of adapting James Lind Alliance methods
Source: Res Involv Engagem. 2026 Nov 9;12:183. doi: 10.1186/s40900-026-01002-3 (PMC13749654; doi:10.1186/s40900-026-01002-3)
Supplement: Supplementary file 2 [file 40900_2026_1002_MOESM2_ESM.pdf]

EVIDENCE SUMMARY – FULL VERSION

Dementia research in the UK has increasingly emphasized the role of cultural context in shaping perceptions of the condition, access to care, and caregiving practices among minority ethnic communities. However, there remains a significant gap in research specifically addressing dementia within the Somali population in the UK. Instead, research has predominantly focused on other ethnic minority groups, particularly South Asian and Black Caribbean populations, whose experiences may provide transferable insights into related cultural and structural barriers. Here, we present a rapid overview of the existing evidence along with a list of potential gaps in the research which merit further investigation.

1. Risk, awareness and prevention

1a. How can awareness of dementia risk / brain health and behaviour change be supported, to reduce risks associated with dementia, in this / similar communities?

WHAT WE KNOW FROM THE RESEARCH
A recent systematic review of qualitative studies (1) explored lay knowledge and beliefs about dementia in Black African communities in the UK, revealing important differences in how the condition was understood.<br><br>Research on other ethnic minority groups (South Asian and Black Caribbean populations) in the UK has revealed that dementia is frequently considered to be a part of normal aging or attributed to supernatural causes, contributing to delays in seeking medical support. In the UK Bangladeshi community (2) cultural perceptions often frame the condition as linked to divine will or moral / spiritual affliction. There have been proposals to involve faith leaders (imams) in tackling these beliefs to reduce stigma as part of the "revivalist Islam" movement.<br><br>Another qualitative study interviewed members of the Pakistani community about their awareness of dementia (3), identifying religious and social barriers to healthcare access.<br><br>In the USA, community-based participatory research (CBPR) has been employed to design dementia education initiatives tailored to African immigrant communities (4, 5) aiming to address misconceptions surrounding dementia, such as its frequent conflation with mental illness or spiritual affliction in some communities.
WHAT WE DON'T KNOW YET: What interventions might be successful for improving awareness of dementia and associated risk factors in the Somali community?
**1b. What interventions have been used to develop, coproduce to adapt the Mediterranean diet for the Somali community?**

WHAT WE KNOW FROM THE RESEARCH

Religious and cultural dietary practices of Somali, Black African, and Muslim communities encompassing Islamic laws (e.g., halal restrictions and fasting rituals) and traditional food choices (e.g., carbohydrate-rich meals and specific animal proteins) may influence brain health and cognitive aging. A 2009 study of Somali individuals in the UK (6) found that their diets were carbohydrate-heavy with limited consumption of fruit and vegetables, which may have implications for cognitive health.

In the USA there have been attempts to promote brain-healthy diets with minorities. For example, the MIND + SOUL pilot study (7) assessed the feasibility of adapting the Mediterranean-DASH Diet Intervention for Neurodegenerative Delay (MIND) to align with African American culinary traditions, incorporating familiar foods, community engagement, and behavioural support.

WHAT WE DON'T KNOW: Can culturally-informed interventions be developed to promote a better diet for brain health with the Somali community?

1c. Have any tools for self-assessment of risks associated with dementia been developed with or adapted for this or 'similar' communities?

WHAT WE KNOW FROM THE RESEARCH

Dementia risk self-assessment tools offer individuals an opportunity to evaluate their dementia risk and engage in preventive strategies. For example, CogDrisk (8) is a general self-assessment tool for dementia risk, incorporating lifestyle, medical, and social risk factors.

Several studies explore cultural adaptation of dementia-related instruments to improve uptake in ethnic minority communities. CognoSpeak (9) is an automated cognitive assessment tool that includes co-development with Somali participants (focused on screening rather than risk prevention). Addenbrooke's Cognitive Examination (ACE-III) has been adapted for British Urdu-speaking populations (10), though again this focused on diagnosis rather than self-assessment.

Other studies look more broadly at adapting dementia prevention messages for ethnically diverse communities (11, 12), including linguistic adjustments and considerations of cultural framing.

WHAT WE DON'T KNOW ABOUT:

The development, cultural adaptation, or testing of self-assessment tools for dementia risk for Somali, Black African, or Muslim communities, with consideration of cultural factors influencing uptake

2. Diagnosis and access

2a. Why do people from this / 'similar' communities access help / diagnosis later? (when experiencing crisis or more severe symptoms)

WHAT WE KNOW FROM THE RESEARCH

Within the UK, minoritised ethnic communities face distinct barriers that contribute to the late presentation of dementia symptoms, delaying formal diagnosis and access to healthcare services. Much of the existing literature centres around Black African-Caribbean or South Asian

communities, highlighting multiple intersecting factors influencing help-seeking behaviours and diagnostic delays, including cultural stigma, religious beliefs, healthcare system distrust and structural inequalities.

A recent qualitative study (13) examined pathways to dementia diagnosis in minoritised UK populations, highlighting how cultural framing and religious identity affect symptom recognition and treatment engagement. Formal dementia services in the UK frequently fail to address Islamic views on family responsibility (birr al-walidayn) and patience (sabr) (14) , contributing to delayed diagnosis and reluctance to seek medical assistance (15, 16).

In Black African and Caribbean communities in the UK (17) researchers found that there was "normalisation" of memory symptoms. It has also been suggested that dementia symptoms may be confused with those of trauma (caused by forced migration). In some minority communities, dementia symptoms are viewed as an inevitable part of aging. There have been attempts to understand cross-cultural differences in symptom interpretation by adapting the Barts Explanatory Model Inventory (BEMI-D) for use in South Asian communities (18, 19).

Structural issues within the healthcare system also play a role. GPs act as gatekeepers to dementia diagnosis, yet disparities between ethnic groups have been noted in referral rates (20, 21) contributing to further mistrust in the health system. Efforts to address these barriers include culturally tailored interventions (22) designed to increase awareness and encourage GP consultations for ethnic minorities. Language barriers and unfamiliarity with UK healthcare pathways compound these challenges, particularly for elderly first-generation migrants with limited English proficiency.

WHAT WE DON'T KNOW YET:

What barriers are faced by Somali patients in accessing GP support for symptoms of memory concerns potentially associated with dementia or cognitive decline?

Effectiveness of interventions to improve dementia diagnostic pathways for people of Somali or other minority ethnic communities.

3. Coping with dementia (for patients / for families)

3a. How can existing evidence / effective ways to manage memory / cognitive / communication difficulties (or other difficulties associated with dementia) be adapted for people from Somali / Black African and Muslim diaspora communities in the UK?

WHAT WE KNOW FROM THE RESEARCH

Recent research has emphasized the necessity of culturally-tailored dementia interventions that reflect the values, caregiving structures, and health perceptions of these communities. Studies highlight barriers to accessing care (23), particularly in Muslim communities, advocating for dementia care models that incorporate Islamic principles, accommodate religious privacy concerns, and engage religious leaders to improve care acceptance (24-26)

WHAT WE DON'T KNOW ABOUT: Interventions for supporting minoritised Muslim communities, including Somali and Black African individuals, in living well with dementia, examining a wide range of outcomes such as quality of life, mood, and independence

3b. What is the experience of caring for elderly first-generation migrants with dementia by their British-born family members?

WHAT WE KNOW FROM THE RESEARCH
The intersection of language barriers, dementia care, and elderly first-generation immigrants in the UK presents a critical challenge within healthcare and social support systems (27, 28). As dementia progresses, multilingual individuals may revert to their native language, complicating communication with healthcare providers who may not be equipped to offer linguistically appropriate care. This issue is particularly pronounced among first-generation immigrants, whose language proficiency in English may already be limited, exacerbating disparities in diagnosis, treatment adherence, and access to support services.<br><br>Caregiving within these communities is often shaped by cultural expectations and religious beliefs. Islamic values foster a sense of filial responsibility, reinforcing familial caregiving roles and strengthening bonds between caregivers and dementia patients (29). Islamic beliefs and rituals, including Quranic recitation and prayer, have been shown to provide emotional comfort to both dementia patients and their caregivers, (27, 16). Studies indicate that Islamic concepts such as **tawakkul** (trust in God), **sabr** (patience), and **thawab** (spiritual reward) play critical roles in helping caregivers manage the psychological stress of care responsibilities (27, 16).<br><br>Research on Arab-American families demonstrates that culturally and linguistically adapted dementia education programs can reduce caregiver burden and improve satisfaction (30, 31).
WHAT WE DON'T KNOW:<br><br>What interventions exist to support family carers from the Somali community manage care for people with dementia living at home? (e.g. peer support or similar activities targeted at women who may become isolated).

References

1. Mokwenye RC. Lay Knowledge and Beliefs Toward Dementia Among the Black African Populations in the UK and Africa: Evidence Synthesis of Qualitative Studies. J Prim Care Community Health. 2024;15:21501319241291786.
2. Hossain MZ, Stores R, Hakak Y, Crossland J, Dewey A. Dementia Knowledge and Attitudes of the General Public among the Bangladeshi Community in England: A Focus Group Study. Dementia & Geriatric Cognitive Disorders 48(5-6):290-296, 2019. 2019;48(5-6):290-6.
3. Nazir N, Kevern P. Understanding and Awareness of Dementia in the Pakistani-Origin Community of Stoke-on-Trent, UK: A Scenario-Based Interview Study. Healthcare (Basel). 2024;12(2).
4. Nkimbeng M, Aswani T, Albers E, Russell W, Shippee T, Gaugler J. Factors associated with knowledge in African immigrants with personal experiences of dementia. Alzheimer's and Dementia:

Diagnosis, Assessment and Disease Monitoring 16(4) (no pagination), 2024 Article Number: e70015 Date of Publication: 01 Oct 2024. 2024;16(4):no pagination.
5. Nkimbeng M, Yam H, Aswani T, Russell WN, Shippee TP, Gaugler JE. Perceptions of Dementia and Dementia Care Among African Immigrants in Minnesota: Insights From Community Conversations. Journals of Gerontology Series B-Psychological Sciences & Social Sciences 79(8), 2024 Aug 01. 2024;79(8).
6. McEwen A, Straus L, Croker H. Dietary beliefs and behaviour of a UK Somali population. Journal of Human Nutrition and Dietetics. 2009;22(2):116-21.
7. Shaw AR, Honea R, Mudaranthakam DP, Young K, Vidoni ED, Morris JK, et al. Feasibility of the MIND+SOUL Culturally Tailored Brain Healthy Diet: A Pilot Study. Am J Lifestyle Med. 2024:15598276241296052.
8. Anstey KJ, Kootar S, Huque MH, Eramudugolla R, Peters R. Development of the CogDrisk tool to assess risk factors for dementia. Alzheimers Dement (Amst). 2022;14(1):e12336.
9. Sahra A, Muse J, Dorota B, Caitlin I, Lise S, Ronan O, et al. Developing an Automated Language-Based Cognitive Assessment; Cognospeak - Working with an under-Represented Ethnic Minority Group. Journal of Neurology, Neurosurgery and Psychiatry Conference: Association of British Neurologists Annual Meeting, ABN 2024 Edinburgh United Kingdom 95(Supplement 2) (pp A35), 2024 Date of Publication: 01 Nov 2024. 2024;95(Supplement 2):A35.
10. Waheed W, Mirza N, Waheed MW, Malik A, Panagioti M. Developing and implementing guidelines on culturally adapting the Addenbrooke's cognitive examination version III (ACE-III): a qualitative illustration. BMC Psychiatry. 2020;20(1):492.
11. Brijnath B, Markusevska S, Lock K, Doherty K, Eccleston C, Navarro Medel C, et al. Evaluating the Effectiveness of a Culturally Adapted Dementia Prevention Animation on Ethnically Diverse participants' Knowledge of Dementia Prevention. Clin Gerontol. 2025:1-14.
12. Brijnath B, Navarro Medel C, Antoniades J, Gilbert AS. Culturally Adapting Evidence on Dementia Prevention for Ethnically Diverse Communities: Lessons Learnt from co-design. Clin Gerontol. 2023;46(2):155-67.
13. Carter C, Roche M, Whitfield E, Budgett J, Morgan-Trimmer S, Zabihi S, et al. Equality of opportunity for timely dementia diagnosis (EQUATED): a qualitative study of how people from minoritised ethnic groups experience the early symptoms of dementia and seek help. Age Ageing. 2024;53(11).
14. Xiao L, Gordon S, Ahmad M. Experiences of family caregivers of people with dementia from a Muslim migrant background in high-income countries: a systematic review and meta-synthesis. Aging Ment Health. 2023;27(12):2319-28.
15. Mukadam N, Cooper C, Basit B, Livingston G. Why do ethnic elders present later to UK dementia services? A qualitative study. Int Psychogeriatr. 2011;23(7):1070-7.
16. Kenning C, Daker-White G, Blakemore A, Panagioti M, Waheed W. Barriers and facilitators in accessing dementia care by ethnic minority groups: a meta-synthesis of qualitative studies. BMC Psychiatry. 2017;17(1):316.
17. Berwald S, Roche M, Adelman S, Mukadam N, Livingston G. Black African and Caribbean British Communities' Perceptions of Memory Problems: "We Don't Do Dementia.". PLoS ONE [Electronic Resource] 11(4):e0151878, 2016. 2016;11(4):e0151878.
18. Kampanellou E, Wilberforce M, Worden A, Giebel C, Challis D, Bhui K. The Barts Explanatory Model Inventory for Dementia: An item reduction approach based on responses from South Asian communities. Int J Geriatr Psychiatry. 2020;35(8):916-25.
19. Giebel CM, Jolley D, Zubair M, Bhui KS, Challis D, Purandare N, et al. Adaptation of the Barts Explanatory Model Inventory to dementia understanding in South Asian ethnic minorities. Aging Ment Health. 2016;20(6):594-602.
20. Ogliari G, Turner Z, Khalique J, Gordon AL, Gladman JRF, Chadborn NH. Ethnic disparity in access to the memory assessment service between South Asian and white British older adults in the United Kingdom: A cohort study. Int J Geriatr Psychiatry. 2020;35(5):507-15.

21. Wilson A, Bankart J, Regen E, Phelps K, Agarwal S, Johnson M, et al. Ethnic variations in referrals to the Leicester memory and dementia assessment service, 2010 to 2017. BJPsych Open. 2020;6(5):e83.
22. Mukadam N, Cooper C, Livingston G. The EAST-Dem study: a pilot cluster randomized controlled trial. Int Psychogeriatr. 2018;30(5):769-73.
23. Hossain MZ, Khan HTA. Barriers to access and ways to improve dementia services for a minority ethnic group in England. J Eval Clin Pract. 2020;26(6):1629-37.
24. Irfan B, Ankouni G, Reader J, Seraji-Bozorgzad N, Giordani B, Bakulski K, et al. Alzheimer's Disease and Related Dementias in Muslim Women: Recommendations for Culturally Sensitive Care. Journal of Alzheimer's Disease 99(3):857-867, 2024. 2024;99(3):857-67.
25. Irfan B, Ankouni G, Reader JM, Seraji-Bozorgzad N, Giordani B, Bakulski KM, et al. Faithful Approaches: Considering Islamic Tenets in ADRD Care for Muslim Women. Alzheimer's & Dementia. 2024;20(S5):e087065.
26. Kevern P, Lawrence D, Nazir N, Tsaroucha A. Religious Influences on the Experience of Family Carers of People with Dementia in a British Pakistani Muslim Community. Healthcare (Basel). 2022;11(1).
27. Chejor P, Laging B, Whitehead L, Porock D. Experiences of older immigrants living with dementia and their carers: a systematic review and meta-synthesis. BMJ Open. 2022;12(5):e059783.
28. Victor CR, van den Heuvel E, Pentecost C, Quinn C, Charlwood C, Clare L. Perspectives of Minority Ethnic Caregivers of People with Dementia Interviewed as Part of the IDEAL Programme. Health & Social Care in the Community. 2024;2024(1):8732644.
29. Daher-Nashif S, Hammad SH, Kane T, Al-Wattary N. Islam and Mental Disorders of the Older Adults: Religious Text, Belief System and Caregiving Practices. Journal of Religion & Health 60(3):2051-2065, 2021 Jun. 2021;60(3):2051-65.
30. Ajrouch KJ, Barada FM, Janevic MR, Antonucci TC. Supporting Arab-American Families Living With Dementia: Testing a Culturally Adapted Program. J Appl Gerontol. 2025;44(4):515-9.
31. Ajrouch KJ, Tarraf W, Brauer S, Zahodne LB, Antonucci TC. Adapted MoCA for Use among Arabic-Speaking Immigrants in the United States. Journal of Cross-Cultural Gerontology 39(4):501-513, 2024 Dec. 2024;39(4):501-13.
