# Supplementary material for: Engaging members of a UK Somali community in identifying dementia research priorities: an example of adapting James Lind Alliance methods
Source: Res Involv Engagem. 2026 Nov 9;12:183. doi: 10.1186/s40900-026-01002-3 (PMC13749654; doi:10.1186/s40900-026-01002-3)
Supplement: Supplementary file 3 [file 40900_2026_1002_MOESM6_ESM.pdf]

WHAT DO WE KNOW ABOUT BRAIN HEALTH?

Introduction

In the UK, scientists are learning that a person's culture affects how they understand dementia, get help, and care for loved ones with the condition - especially in communities from minority ethnic backgrounds. However, there has been very little research about how dementia affects the Somali community in the UK – most studies look at other groups, like people from South Asian or Black Caribbean backgrounds. There have also been studies of African immigrants living in the USA. These contexts are different, but they might give us some clues about the challenges faced. This summary provides a quick overview of what we already know, and points out important areas where more research is needed.

THEME 1a: Awareness & risk

WHAT WE KNOW FROM THE RESEARCH
Researchers asked people from different ethnic minorities about their views and knowledge of dementia. They found that these groups often understand dementia differently than most doctors do.<br><br>Studies show that people from South Asian and Black Caribbean backgrounds in the UK often think dementia is just a normal part of getting old, or that it's caused by magic or spirits. Because of these beliefs, they might wait too long to go to the doctor.<br><br>In the Bangladeshi community, some people think dementia is linked to God's will, or that it's a punishment for bad behaviour, leading to **stigma** and **shame** for those affected. Some people have suggested that religious leaders (imams) could help change these beliefs and reduce the shame around dementia. Another study talked to people from the Pakistani community and found that their belief in family responsibility can make it hard for them to get the healthcare they need for dementia.<br><br>In the USA, researchers have worked directly with African immigrant communities to create special programs that teach them about dementia. These programs try to clear up misunderstandings and encourage people to seek help
WHAT WE DON'T KNOW YET: How could make people in the Somali community more aware of dementia and its risks?

1b: Diet

WHAT WE KNOW FROM THE RESEARCH: The foods people eat can affect their brain health as they get older. This is especially true for people from Somali, Black African, and Muslim communities, where religious rules and cultural traditions play a big role in what they eat.

Religious rules: Islamic laws, like halal restrictions and fasting during Ramadan, can affect what kinds of foods are eaten and when.

Cultural traditions: A study in 2009 looked at Somali people living in the UK and found that their diets often had a lot of carbohydrates but not many fruits and vegetables. This kind of diet might not be the best for brain health.

In the USA, there have been some programs are trying to help minority communities improve what they eat to keep their brain healthy. For example, a project called MIND + SOUL adapted the Mediterranean diet to include foods that are familiar to African Americans. This project also involves the community and provides support to help people change their eating habits.

WHAT WE DON'T KNOW:

Could we help Somali people to eat a more "brain healthy" diet by recommending foods that would be familiar to their community?

1c: Reducing your risk

WHAT WE KNOW FROM THE RESEARCH

There are **tests** people can take to see how likely they are to develop dementia. These tests can also help people figure out what they can do to lower their risk.

CogDrisk: This test looks at your lifestyle, medical history, and social life to estimate your dementia risk. However, it's not tailored to ethnic minorities and that might mean it's not very appealing to them. Scientists are working on making these kinds of tests easier to use to encourage people from different cultural backgrounds to try them.

CognoSpeak: This is a computer-based test for checking someone's brain function. It was developed with help from Somali people, but it's used to see if someone already has dementia, not to predict their risk.

Addenbrooke's Cognitive Examination (ACE-III): This is another test for diagnosing dementia, and it's been adapted for people who speak Urdu in the UK. Again this is for diagnosis of dementia, rather than risk.

WHAT WE DON'T KNOW:

Could adapting a risk assessment tool like CogDrisk help more Somali people reduce their risk of dementia?

THEME 2: Diagnosis

WHAT WE KNOW FROM THE RESEARCH:

In the UK, people from minority ethnic groups often seek help for dementia much **later** than other people. Previously, researchers have looked at why this is the case for Black African-Caribbean and South Asian communities, and found there were reasons such as:

Cultural stigma: People might feel ashamed or embarrassed about dementia.

Religious beliefs: Some people believe dementia is an evil spirit or a punishment from God, rather than an illness.

Distrust of the healthcare system: Some people don't trust doctors or hospitals, or believe that it is the duty of their family to take care of them.

Structural inequalities: This means that the system itself isn't fair to everyone.

Recent studies have shown that culture and religion affect how people see dementia symptoms and whether they seek treatment. In Black African and Caribbean communities, people sometimes think memory problems are just a normal part of getting older. Sometimes, the symptoms of dementia might be confused with the effects of trauma, especially if someone has experienced forced migration. Scientists are trying to understand how different cultures view dementia symptoms. One tool they're using is the Barts Explanatory Model Inventory (BEMI-D), which they've adapted for use in South Asian communities.

The healthcare system itself also creates problems. For example, doctors (GPs) are the ones who usually refer people for dementia diagnosis, but studies have shown that they don't refer people from minority ethnic groups as often. This could be for a number of reasons, but it makes people trust the system even less.

To try and fix these problems, some groups are creating special programs that are designed to be sensitive to the needs of different cultures. These programs aim to teach people about dementia and encourage them to see their doctor. Language barriers and not knowing how the UK healthcare system works also make it harder, especially for older people who moved here later in life and don't speak much English.

WHAT WE DON'T KNOW YET:

What are the barriers for Somali patients and their families in seeking help for memory problems or brain health?

How can we make it easier for people of Somali or other minority communities to see a specialist to find out if they have dementia?

THEME 3: Coping with dementia

3a: For patients

WHAT WE KNOW FROM THE RESEARCH
It's difficult for people from ethnic minorities to find dementia care that properly understands and respects their culture and religion. This can be particularly challenging for women, who have specific beliefs about things like dressing modestly (maybe wearing a hijab or niqab), privacy, and their role in the family.<br><br>Dementia care needs to be more inclusive of these communities to allow those affected (and their families) to lead better lives.
WHAT WE DON'T KNOW: How can we provide culturally-sensitive care to Somali people so that they can live well with dementia?

3b: For families:

WHAT WE KNOW FROM THE RESEARCH
In many of these communities, **family members** take care of their loved ones with dementia because of their culture and religion. This creates strong bonds between the person with dementia and their family. However, it can sometimes put a strain on families if they don't have any other support.<br><br>Islamic ideas like **tawakkul** (trusting in God), **sabr** (being patient), and **thawab** (spiritual reward) help family members cope with the stress of caregiving. Reading the Quran and praying can provide comfort to both the person with dementia and the person taking care of them.<br><br>However, in the UK, there can be **communication problems** when older people who moved from other countries get dementia, especially if they don't speak much English. As dementia gets worse, people might forget their second language (English) and only remember their first language. This means doctors and nurses who don't speak that language can't communicate with them. And if their children were born in the UK, even they might struggle to understand them. This can lead to them not getting diagnosed properly, not taking their medicines correctly, and not getting the support they need.
WHAT WE DON'T KNOW:<br><br>What support do Somali family caregivers need to prevent them feeling isolated and help them look after their loved ones?
