# Supplementary material for: Engaging members of a UK Somali community in identifying dementia research priorities: an example of adapting James Lind Alliance methods
Source: Res Involv Engagem. 2026 Nov 9;12:183. doi: 10.1186/s40900-026-01002-3 (PMC13749654; doi:10.1186/s40900-026-01002-3)
Supplement: Supplementary file 4 [file 40900_2026_1002_MOESM10_ESM.docx]

**Phase 3, Questions taken to Workshop 2 for prioritising**

NB: the process of identifying the questions for Phase 3 was driven by the need to communicate questions in a way participants could understand and to create questions for prioritising from the evidence briefing.

|  | **Category for voting cards and to aid discussion** | **Question** |
| --- | --- | --- |
| Q1 | *Awareness of dementia and risk* | How do people in the UK Somali community understand dementia and brain health? |
| Q2 | *Awareness of dementia and risk* | How can people in the UK Somali community be supported to become more aware of what dementia is? |
| Q3 | *Awareness of dementia and risk* | How can people in the UK Somali community be supported to become more aware of what can increase the risks of developing dementia? |
| Q4 | *Reducing risk* | How can people in the UK Somali community be supported to keep their brains as healthy as possible?  (on back of card: and so, potentially, reduce risk of developing dementia or worsening cognitive decline (thinking skills like memory, concentration, planning and organising) BUT important when we talk about risk - chance - it's about reducing, can never completely get rid of it. Sadly, people can be very healthy and still get dementia ; we can’t say one or more specific factors (e.g. smoking, diet) CAUSE dementia and keeping brain healthy is likely to reducing risks associated with other conditions e.g heart disease, stroke or diabetes |
| Q5 | *Diet (improving brain health & reducing dementia risk)* | Does the diet of the UK Somali Community affect brain health and dementia risk, if so how? |
| Q6 | *Diet (improving brain health & reducing dementia risk)* | How could the UK Somali community be supported to eat a brain healthy diet? |
| Q7 | *Diet (improving brain health & reducing dementia risk)* | Can foods and ways of cooking or preparing food that are familiar to, and culturally appropriate for the UK Somali community, be recommended for brain health? |
| Q8 | *Assessing risk* | Can ways to assess risks linked to developing dementia be developed with, or adapted for, the UK Somali community, or other minority communities?  (on back of card:important when we talk about risk - chance - it's about reducing, can never completely get rid of it. Sadly, people can be very healthy and still get dementia ; we can’t say one or more specific factors (e.g. smoking, diet) CAUSE dementia. Also keeping our brain healthy is likely to reduce risks associated with other conditions e.g heart disease, stroke or diabetes |
| Q9 | *Assessing risk* | Could ways that we already have, to help people assess risk of developing dementia help people in the UK Somali community (and other communities) become more aware of their risk?  Back of card:i.e. Would using existing tools help improve awareness of risk factors for this community(awareness different to changing behaviour)  important when we talk about risk - chance - it's about reducing, can never completely get rid of it. Sadly, people can be very healthy and still get dementia ; we can’t say one or more specific factors (e.g. smoking, diet) CAUSE dementia. Also keeping our brain healthy is likely to reduce risks associated with other conditions e.g heart disease, stroke or diabetes |
| Q10 | *Assessing risk* | What support can be offered to people from the UK Somali community (or other communities) if they find out they have risks associated with developing dementia?  (on back of card: i.e. if told we have increased risk , what help and support can or should be offered - to help people improve their brain health ;  important when we talk about risk - chance - it's about reducing, can never completely get rid of it. Sadly, people can be very healthy and still get dementia; we can’t say one or more specific factors (e.g. smoking, diet) CAUSE dementia. Also keeping our brain healthy is likely to reduce risks associated with other conditions e.g heart disease, stroke or diabetes |
| Q11 | *Getting help with memory problems, dementia diagnosis* | How can services make it easier for people from the Somali (or other minority communities) to see a specialist to find out if they have dementia or not? |
| Q12 | *Getting help with memory problems, dementia diagnosis* | How can the UK Somali community (or other minority communities) be supported to see a specialist to find out if they have dementia or not? |
| Q13 | *Living with dementia, coping and caring* | What does living well (good quality of life) with dementia mean to people in the UK Somali community? |
| Q14 | *Living with dementia, coping and caring* | What would good care for people living with dementia in the UK Somali community look like?  Back of card: can be any care setting  what would it involve? What would culturally appropriate/sensitive care look like |
| Q15 | *Living with dementia, coping and caring* | What would good support for family carers of people living with dementia in the UK Somali community look like?  Back of card: for people caring at home but could be in other settings e.g. day care  what would it involve? What would culturally appropriate/sensitive care look like |
| Q16 | *Living with dementia, coping and caring* | What support do Somali family carers of people living with dementia need to prevent them feeling isolated? |
| Q17 | *Living with dementia, coping and caring* | Can faith-based activities or being part of a faith based community, reduce the impact of dementia on people with dementia, and their family carers?  Back of card: like going to mosque, praying, routines of reading the Quran; there is some evidence - Islamic beliefs and rituals have been shown to provide emotional comfort to people with dementia and their caregivers e.g. trust in God (tawakkul), patience (sabr). Thawab (spiritual reward) but only some evidence |
