# Supplementary material for: Engaging members of a UK Somali community in identifying dementia research priorities: an example of adapting James Lind Alliance methods
Source: Res Involv Engagem. 2026 Nov 9;12:183. doi: 10.1186/s40900-026-01002-3 (PMC13749654; doi:10.1186/s40900-026-01002-3)
Supplement: Supplementary file 5 [file 40900_2026_1002_MOESM1_ESM.docx]

**Members of the project management group:**

[Dr Dan Blackburn, (Consultant Neurologist, Researcher), University of Sheffield](https://www.sheffield.ac.uk/smph/people/neuroscience/daniel-blackburn)

Ismail Yussuf, Finance and Operations Director, Israac

[Dr Sam Creavin, (GP, Researcher) University of Bristol](https://www.bristol.ac.uk/people/person/Sam-Creavin-7a04c325-7a39-4560-8308-8cbcaa763747/)

[Dr Josie Reynolds (GP, Researcher), University of Sheffield](https://sites.google.com/sheffield.ac.uk/dera/home/dera-team#h.2bf72fjyjgv9)

[Professor Alicia O’Cathain (Researcher), University of Sheffield](https://www.sheffield.ac.uk/smph/people/population-health/alicia-ocathain)

Lise Sproson (Patient and Public Involvement expert, Devices for Dignity National Institute of Health)

[Kate Gridley (Researcher), University of York](https://www.york.ac.uk/spru/staff/kate-gridley/)

[Dr Sarah Barnes (Researcher), University of Sheffield](https://www.sheffield.ac.uk/smph/people/population-health/sarah-barnes)

Sahra Abdi Community Research Link Worker, Israac

Muse Jama Community Research Link Worker, Israac
