# Supplementary material for: Engaging members of a UK Somali community in identifying dementia research priorities: an example of adapting James Lind Alliance methods
Source: Res Involv Engagem. 2026 Nov 9;12:183. doi: 10.1186/s40900-026-01002-3 (PMC13749654; doi:10.1186/s40900-026-01002-3)
Supplement: Supplementary file 6 [file 40900_2026_1002_MOESM3_ESM.docx]

**Phase 2, Rapid evidence briefing search strategy**

| **POPULATION 1** | **POPULATION 2** | **CONCEPT 1** | **CONCEPT 2** | **CONTEXT** |
| --- | --- | --- | --- | --- |
| Somali community  Black African  Muslim | Dementia  Alzheimer’s  Brain health | Prevention | Diet / nutrition  (& interventions to adapt) | UK + international/global |
|  |  |  | Self-assessment of risk  (& uptake of this) |  |
|  |  | Diagnosis | Late presentation |  |
|  |  |  | Barriers to GP access |  |
|  |  | Caregiver support | Language / communication |  |
|  |  | Faith | |  |
| Somali community | | Cultural adaptation of (any) interventions | |  |
| Dementia | |  |  |  |
