# Supplementary material for: Engaging members of a UK Somali community in identifying dementia research priorities: an example of adapting James Lind Alliance methods
Source: Res Involv Engagem. 2026 Nov 9;12:183. doi: 10.1186/s40900-026-01002-3 (PMC13749654; doi:10.1186/s40900-026-01002-3)
Supplement: Supplementary file 7 [file 40900_2026_1002_MOESM4_ESM.docx]

**Phase 1 - Round 2 of reformatted questions,** after feedback from project management group, on Round 1 questions, shared with project management team, and then shared with information specialist as draft questions to guide rapid evidence search

|  | **Initial Phase 1 theme** | **Draft questions** |
| --- | --- | --- |
| 1 | Prevention | “What is known about Somali/black African/similar diets and brain health and/or dementia risk?”  + /or  “What examples or evidence are there of interventions that develop, adapt, coproduce and test out / trial adaptations to the mediterranean diet to suit particular communities?”  (community mentioned sugar and carbs, diabetes specifically) |
| 2 | Prevention | How can awareness of dementia risk / brain health and behaviour change be supported, to reduce risks associated with dementia, in this / similar communities? |
| 3 | Knowledge and  Awareness | Have any tools for self-assessment of risks associated with dementia been developed with or adapted for this or ‘similar’ communities  (or join with above/ could cut) |
| 4 | Knowledge and awareness | What strategies/ interventions have been implemented effectively to promote dementia awareness within minority ethnic communities? |
| 5 | Access | Why do people from this / ‘similar’ communities access help / diagnosis later (when experiencing crisis or more severe symptoms) |
| 6 | Access | What are the experiences of this / other communities with accessing home care packages / support after diagnosis, as dementia progresses? + Any evidence of effective interventions/ services? |
| 7 | Access | What are (suggested?) reasons for blocks that racially minoritised communities experience when presenting at the GP / primary care with memory concerns. |
| 8 | Access | Have an interventions or care pathways been (co) designed to improve diagnostic pathway in primary and secondary care for people from this / similar communities  (or join up with above question)? |
| 9 | Treatment | How can existing evidence / effective ways to manage memory / cognitive / communication difficulties (or difficulties associated with dementia be adapted for people from this/ ‘similar’ communities    + / OR  What evidence is there about how to manage and cope with (symptoms/challenges cognitive changes) dementia for people from similar / ethnic minority communities |
| 10 | Care | Is there (any) evidence about family carers not speaking the same language as people with dementia, when due to dementia the person with dementia may revert back to their ‘mother tongue’ (first language, home language?) yet family carers living or born in UK may not speak that language fluently-and the subsequent impact and any coping strategies? |
| 11 | Care | Is there any evidence about key components of ‘culturally appropriate’ dementia support (what it would ‘look like’)? |
| 12 | Care | How can faith (faith-based activities/being part of faith-based community) reduce symptoms of dementia / strengthen care-partner bond, reduce stress / increase wellbeing of persona with dementia and or carer? |
| 13 | Care | Have existing approaches / interventions to support people to live well with dementia been adapted or used within this or similar communities (such as self-help self-management peer support groups, intervention outcomes such as QoL, satisfaction, improved mood, sense of competence, hope, independence, function)? |
| 14 | Care | What interventions exist to support family carers from this / ‘similar’ communities manage care for people with dementia living at home?  (intervention outcomes e.g. information based/ other outcomes such as wellbeing or coping, stress, QoL) |
| 15 | Stigma and isolation | What effective peer support interventions addressing dementia carer isolation and or stigma that could be adapted for this community? |
| 16 | Stigma and isolation | Are there effective interventions for isolated women carers or people with dementia (that could be adapted to the needs of women in this community)? |
| 17 | Stigma and isolation | Have there been any effective interventions to address dementia stigma and isolation in minority ethnic / Somali communities? That could be adapted for communities such as this  Possibly cut this question, does it duplicate? |
