# Supplementary material for: Engaging members of a UK Somali community in identifying dementia research priorities: an example of adapting James Lind Alliance methods
Source: Res Involv Engagem. 2026 Nov 9;12:183. doi: 10.1186/s40900-026-01002-3 (PMC13749654; doi:10.1186/s40900-026-01002-3)
Supplement: Supplementary file 8 [file 40900_2026_1002_MOESM5_ESM.docx]

**Phase 4, the top 10 questions taken to Workshop 3 -** as derived from voting tallies in Workshop 2 (in which participants had voted with stickies on the 17 questions presented to them in Workshop 2)

|  | ***Category for cards and to aid discussion*** | | | **Question** |
| --- | --- | --- | --- | --- |
| Q1  (Workshop 2 -Q1) | *Awareness (of dementia and risk)* | | | How do people in the UK Somali community understand dementia and brain health? |
| Q2  (Workshop 2- Q2) | *Awareness of dementia and risk* | | | How can people in the UK Somali community be supported to become more aware of what dementia is? |
| Q3  (Workshop 2- Q5) | *Diet (improving brain health & reducing dementia risk)* | | | Does the diet of the UK Somali Community affect brain health and dementia risk, if so, how? |
| Q4  (Workshop 2- Q7) | | *Diet (improving brain health & reducing dementia risk)* | Can foods and ways of cooking or preparing food that are familiar to, and culturally appropriate for the UK Somali community, be recommended for brain health? | |

| Q5  (Workshop 2- Q11) | *Getting help with memory problems, dementia diagnosis* | How can services make it easier for people from the Somali (or other minority communities) to see a specialist to find out if they have dementia or not? |
| --- | --- | --- |
| Q6  (Workshop 2-Q12) | *Getting help with memory problems, dementia diagnosis* | How can the UK Somali community (or other minority communities) be supported to see a specialist to find out if they have dementia or not? |
| Q7  (Workshop 2-Q13) | *Living with dementia, coping and caring* | What does living well (good quality of life) with dementia mean to people in the UK Somali community? |
| Q8  (Workshop 2-Q14) | *Living with dementia, coping and caring* | What would good care for people living with dementia in the UK Somali community look like?  Back of card: can be any care setting  what would it involve? What would culturally appropriate/sensitive care look like |

| Q9  (Workshop 2- Q16) | *Living with dementia, coping and caring* | What support do Somali family carers of people living with dementia need to prevent them feeling isolated? |
| --- | --- | --- |
| Q10  (Workshop 2-Q17) | *Living with dementia, coping and caring* | Can faith-based activities or being part of a faith based community, reduce the impact of dementia on people with dementia, and their family carers?  Back of card: like going to mosque, praying, routines of reading the Quran; there is some evidence - Islamic beliefs and rituals have been shown to provide emotional comfort to people with dementia and their caregivers e.g. trust in God (tawakkul), patience (sabr). Thawab (spiritual reward) but only some evidence |
