# Supplementary material for: Engaging members of a UK Somali community in identifying dementia research priorities: an example of adapting James Lind Alliance methods
Source: Res Involv Engagem. 2026 Nov 9;12:183. doi: 10.1186/s40900-026-01002-3 (PMC13749654; doi:10.1186/s40900-026-01002-3)
Supplement: Supplementary file 9 [file 40900_2026_1002_MOESM7_ESM.docx]

**Phase 2, MEDLINE/Embase/PsycINFO search strategy** (searched 13/3/25)

1	*africa/ or *"africa south of the sahara"/ or *africa, eastern/ or somalia/

2	exp "emigrants and immigrants"/ or refugees/

3	somali*.mp.

4	(black african* or muslim).mp.

5	1 or 2 or 3 or 4

6	exp Dementia/

7	(dementia or alzheimer* or brain health).mp.

8	6 or 7

9	3 and 8

10	("culturally specific" or (cultur* adj3 (appropriate or adapt* or tailor* or modif*))).mp.

11	(prevention or risk factor* or risk assessment or self-assessment or diet* or nutrition* or food* or eating).mp.

12	(late present* or diagnos* or GP or general practi*).mp.

13	((family or families or relatives or relations* or partner*) adj3 (carer* or caring or caregiver*)).mp.

14	(islam* or religio* or faith or faiths or belief* or spiritual*).mp.

15	5 and 8 and 10

16	5 and 8 and 11

17	5 and 8 and 12

18	5 and 8 and 13

19	5 and 8 and 14

20	9 or 15 or 16 or 17 or 18 or 19

Results following deduplication: **827**
