# Supplementary material for: Engaging members of a UK Somali community in identifying dementia research priorities: an example of adapting James Lind Alliance methods
Source: Res Involv Engagem. 2026 Nov 9;12:183. doi: 10.1186/s40900-026-01002-3 (PMC13749654; doi:10.1186/s40900-026-01002-3)
Supplement: Supplementary file 10 [file 40900_2026_1002_MOESM8_ESM.docx]

**Phase 4, visual summary of discussions in Workshop 3**

**Note:** These visual summaries were completed live (‘visual scribing’) during Workshop 3. As most of the discussion centred around questions 8 and 10, that is what is summarised and captured here. The live illustration process was intended as a summary to share key points with participants on the day and to ask them if they felt they captured the main points of the discussion. When participants were asked this, they said they said they felt they captured the main discussion points. The visual scribing process was not intended as a detailed record of all discussions.
