# Supplementary material for: Engaging members of a UK Somali community in identifying dementia research priorities: an example of adapting James Lind Alliance methods
Source: Res Involv Engagem. 2026 Nov 9;12:183. doi: 10.1186/s40900-026-01002-3 (PMC13749654; doi:10.1186/s40900-026-01002-3)
Supplement: Supplementary file 11 [file 40900_2026_1002_MOESM9_ESM.docx]

**Phase 1, Round 1 of reformatting questions**, from questions and comments expressed in Workshop 1, shared with project management group (PMG)

**Table 1 Prevention**

|  | **Prevention (James Lind Alliance category)** | **Example comment from workshop notes**) |
| --- | --- | --- |
| 1 | Can dementia onset be prevented or delayed by dietary or nutritional factors (JLA question)  [re: general population] | *Can the diet we eat contribute to dementia?* |
| 2 | How can members of this community/’similar’ be supported to understand their risks of developing dementia?  What evidence is there of effective interventions to do this | *What prevents dementia?*  *Understanding our risks of developing dementia* |
| 3 | Could the Somali (or ‘similar’) diet be modified to support brain health? (including sugar and carbs specifically?) | *High intake of sugar/carbs*  *Very sweet tea at cafes* |
| 4 | Are there ways individuals can assess their individual dementia risk?  OR  [have any tools for self-assessment of dementia been developed with or adapted for this or ‘similar’ communities?) | *How does my particular lifestyle affect my brain and how can I prevent it?* |

**Table 2 Diagnosis**

|  | **Diagnosis (James Lind Alliance category)** | Example comment |
| --- | --- | --- |
| 5 | Why do people from this / ‘simi  lar’ communities access help /diagnosis later when people are experiencing the more severe stages of dementia? | *understanding why our community access help late?* |
| 6 | How do people (and/or families) with dementia know they have dementia?  or  What should people [from this / ‘similar’ community] do if they think they have memory difficulties or dementia? | *Recognising the problem* |
| 7 | Can the difference between dementia and other / normal age related memory problems be distinguished? (taken from JL) | *What is the difference between normal forgetfulness and dementia?* |
| 8 | Is there a way for individuals to know / diagnose dementia themselves (suitable for ethnic minorities/non English speakers/ Somali)? | *How would I know if I had dementia?* |

**Table 3 Treatment**

|  | **Treatment (James Lind Alliance category)** | Example comment |
| --- | --- | --- |
| 9 | How should we respond to people with dementia if they are disorientated in time? | *Displaced in time* |
| 10 | What are most effective ways to manage memory and communication difficulties (or cognitive difficulties inc. disorientation in time?) associated with dementia (all stages) for people from this/ ‘similar’ communities | *Memory / conversation makes group conversation difficult* |
| 11 | How do language barriers for people with dementia (from this community/ ‘similar’) impact participation in possible activities and interaction with (younger) members / carers/ family - don’t speak Somali as well / at all?  OR  What are the best ways to facilitate engagement in activities for non-English speakers with dementia/memory difficulties in the UK? | *Language barrier prevents participation in possible activities* |
| 12 | What is culturally appropriate dementia support?  OR  What would / could culturally appropriate dementia support look like for this / ‘similar’ community? Examples of good practice/recommendations? | *Culturally appropriate dementia support* |
| 13 | Can people use spiritual daily activities to help manage memory problems/ cope with symptoms? | when I have problems with remembering and health issues, I read the Quran |

**Table 4 Care**

|  | **Care (at home/in community; and carers)**  **(James Lind Alliance category)** | Example comment |
| --- | --- | --- |
| 14 | How do we get a care package/support reviewed ->  What are the best ways of monitoring and assessing the need for formal, paid care/changes to care packages (models for doing this especially within minority communities?) | Gran has dementia but NHS don't answer phone calls and granddaughter feels family have to do everything request for more care denied |
| 15 | What are the best ways / recommended ways to live a good life with dementia? | Can you live with dementia and still have a good life? |
| 16 | How can we better support Somali families who are caring?  [What interventions exist to support family carers from this / ‘similar’ communities? What helps families manage care at home?] | Lack of carer support  I need help to care, carers can't  come overnight, have problems myself, need help |
| 17 | What kind of information would best support Somali or ‘similar’ carers (of people with dementia)? | Info for families about how to care |
| 18 | Can peer discussion/ support help raise awareness or help carers feel less isolated in this /‘similar’ communities?) OR  What is the role of peer support to help the challenges of dementia / stigma/ isolation in these community / are there effective interventions…? | It's good to share experiences and groups like this help with that |
| 19 | Are there effective interventions to reduce isolation for esp women carers of people with dementia (from this / community / ‘similar’ )? | Lack of socialisation, 'isolation' especially for women - often stay at home -> better spaces for women socialisation without men |

**Table 5 Knowledge and awareness**

|  | **Additional category - knowledge & awareness** | Example comments |
| --- | --- | --- |
| 20 | What are the best strategies to raise awareness & knowledge about dementia and brain health? For members of minority ethnic communities in the UK, particularly those of Somali heritage / black African / Muslim backgrounds?  [who it affects, possible treatments, risk reduction) OR  What strategies/ interventions have been implemented effectively to promote dementia awareness within minority ethnic communities? | Can you get it any age?  Where can we get more information about dementia?  Some myths and mis-info like tap water causes dementia  What is the difference between forgetfulness and dementia?  Understanding more about dementia: what it is? How it affects the brain? Possible treatments? |
| 21 | Why does this community /’similar’ experience inequalities in terms of dementia (access/ diagnosis, care, treatment, risk factors?) | Understanding inequalities in Somali community re: dementia |

**Table 6: Stigma and Isolation**

|  | **Additional category: Stigma & isolation** | Example comments |
| --- | --- | --- |
| 22 | What can help reduce dementia stigma in this community? / What has worked with other underserved /minority communities? OR  Are there effective interventions to address dementia stigma and isolation in minority ethnic / Somali communities? Esp for women carers?(from communities such as this/ BAME)? | Sometimes we are in the same 4 walls all the time, we don't go out and see people / exercise  Shame/stigma/phobia to reveal diagnosis -> shunned from community |
| 23 | What can encourage people from this / ‘similar’ community to seek help? | not help seeking because of stigma |
| 24 | Can people with dementia still work and earn money? | People work into old age so dementia could ruin their livelihoods and people more likely to stay home / stop doing things, don't want to be caught out |

**Table 7: Access**

|  | **Additional category: access** | Example comments |
| --- | --- | --- |
| 25 | How can we encourage people from this / ‘similar’ community to go to primary care with memory concerns [when they report negative experiences re: trying to access primary care generally?] or are there any (effective) interventions that encourage this? | When I went to Dr's for painkillers, they told me to go buy a tablet, so why would I bother going to the DR? |
| 26 | How can primary care support a more effective diagnosis service for people from this community / ‘similar’ communities? | I know if we have these problems, we should go to the Dr, but if we cannot get appointment for other health problems, why would we go with this? We just put up with it |
| 27 | How can primary and secondary care improve access for Somali community /'similar ' communities?  OR  [Why don't we see Somali people in secondary care (memory services)?  OR  What are primary care/GP referral rates to memory services for people from this / 'similar' communities, how do they compare to rates for the majority/English speaking/heritage population? What are the reasons for that?] | Why don't we see Somali people in secondary care? Participant says they DO go to GP but aren't referred on |

**Table 8 Examples of exclusions**

| **Example comment** | **Reason for exclusion** |
| --- | --- |
| At home everyone knew everything, no secrets. Here culture is keep to yourself, You get depressed. | No underlying research question |
| I got problem, put the pan on fire, and I forgot  Can forget how many times prayed that day | No underlying research question |
| It is very essential to improve how to promote the access and support to the concerned department for the communities | No underlying research question |
| I am worry that I could have dementia what can I do? | No underlying research question request for info |
| Does not sleeping cause dementia? | Cause related - out of scope |
| Does technology/mobile phones contribute to cognitive decline? | Cause related - out of scope |
